# De novo promoters emerge more readily from random DNA than from genomic DNA

**DOI:** 10.1126/sciadv.aec2554

**Published:** 2026-05-29

**Authors:** Timothy Fuqua, Andreas Wagner

**Affiliations:** ^1^ Department of Evolutionary Biology and Environmental Studies, University of Zurich, Zurich, Switzerland.; ^2^ Swiss Institute of Bioinformatics, Quartier Sorge-Batiment Genopode, Lausanne, Switzerland.; ^3^ Institute of Ecology and Evolution, University of Bern, Bern, Switzerland.; ^4^ The Santa Fe Institute, Santa Fe, NM, USA.

## Abstract

Promoters are DNA sequences that initiate transcription. We studied the propensity of both random and genomic DNA to initiate transcription. To this end, we assayed the promoter activity of 17,129 random, synthetic DNA sequences and 91,866 *Escherichia coli* genomic sequences. Genomic DNA encodes ~1.3× more promoters than random DNA. This higher incidence of promoters also holds for intragenic regions, suggesting an underappreciated role for intragenic promoters. We also explored the propensity of nonpromoter sequences to become promoters. To this end, we chose 225 random and 60 genomic sequences without promoter activity, created over half a million DNA mutants from them, and assayed these mutants for novel promoter activity. Promoters emerge ~3× more readily from random DNA than from genomic DNA, because the genome contains fewer proto-binding sites for transcriptional activators. Our work shows that the genome has a smaller evolutionary potential to create new transcripts than random DNA.

## Teaser

Promoters emerge three times more readily from random DNA than from genomic DNA.

## INTRODUCTION

Gene regulation enables organisms to respond to their environment (*1*). It is controlled at the level of transcription by promoter DNA sequences (*2*). Prokaryotic promoters encode binding sites for sigma (σ) factors that help RNA polymerase bind near a gene (*3*–*6*). Promoters may also encode binding sites for transcription factors (TFs) to activate or repress transcription (*1*, *7*).

Mutations in promoters can have profound impacts on their activity (*7*–*12*). For example, mutations can create new transcriptional activity by destroying a binding site for a TF that represses transcription (*7*, *13*, *14*). In addition, mutations can create new promoters by creating new binding sites for either a σ factor or a TF that activates transcription (*13*–*17*). The resulting de novo promoters can create evolutionary innovations, either by changing the regulation of existing genes (*18*–*20*) or by creating de novo transcripts (*21*, *22*).

Approximately 10% of synthetic random DNA sequences have promoter activity (*15*, *17*, *23*, *24*), which shows that prokaryotic promoters are simple DNA sequences likely to arise by chance alone. In contrast to synthetic random DNA, genomes have been subject to millions of years of evolution (*25*, *26*). During this time, natural selection has helped to create and preserve promoters to transcribe protein coding genes. At the same time, natural selection has also acted against promoters that coincidentally exist inside of coding DNA (*27*–*29*). For example, computational analyses reveal that the *Escherichia coli* genome overall contains fewer promoter-like sequences than random DNA (*15*, *17*, *30*), and its protein-coding DNA is impoverished in codons that resemble parts of σ factor binding sites (*11*, *30*). Thus, there are simultaneous selective pressures to preserve or improve the promoters of thousands of functional genes (*31*) and also to remove promoters inside these genes (*32*–*34*). This dichotomy may influence how new promoters emerge in the *E. coli* genome.

Here, we experimentally explore the propensity of both random and genomic DNA to be promoters by assaying over 100,000 DNA sequences for promoter activity. We also explore the propensity of such nonpromoter sequences to become promoters by assaying mutagenesis libraries from 225 transcriptionally inactive genomic sequences and 60 inactive random synthetic sequences without promoter activity.

## RESULTS

### Random DNA is very likely to encode a promoter, and genomic DNA even more so

**Fig. 1.**
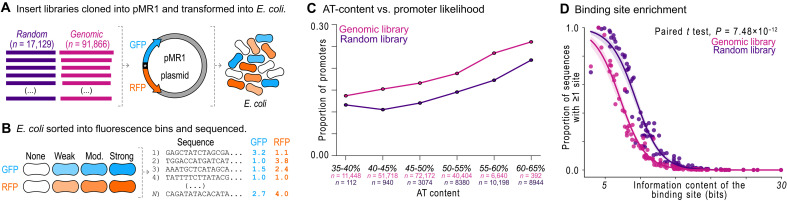
The genome is enriched with active promoters relative to random DNA. (**A**) We cloned the random library of 150-bp N-mer sequences and the genomic library of 100- to 300-bp sequences into the dual-reporter plasmid MR1 (pMR1), which drives the expression of green fluorescent protein (GFP; teal) from inserts on the top DNA strand, and that of red fluorescent protein (RFP; orange) on the bottom strand. We transformed *E. coli* cells with the plasmid libraries. (**B**) We sorted the bacterial libraries into fluorescence bins: none, weak, moderate, and strong for both GFP and RFP with a cell sorter. We bulk sequenced the library inserts from each bin and calculated fluorescence scores in arbitrary units (a.u.) (Materials and Methods). (**C**) The probability that a DNA sequence in the random and genomic libraries is a promoter relative to its AT content. (**D**) For 102 position weight matrices (PWMs) for TFs and σ factors, we plot the percentage of sequences in each library that encode at least one predicted binding site (vertical axis) against the respective PWM’s information content in bits. We test for equality of the frequency distributions between the random and genome libraries with a paired *t* test (*P* = 7.48 × 10^−12^). See Source Data.

We first asked how often random DNA encodes a bacterial promoter compared to genomic DNA. To answer this question, we created a random DNA library by synthesizing a pool of oligonucleotides (IDT, USA), each 150 base pairs (bp) in length, where each position has a 25% chance of encoding an A, T, C, or G (Fig. 1A). Second, we created the genomic DNA library by fragmenting the *E. coli* genome into 100- to 300-bp pieces using ultrasonication (Materials and Methods). We cloned these libraries into a dual-reporter plasmid (*35*) (pMR1) between a green (red) fluorescent protein coding sequence downstream (upstream) of a library sequence and transformed *E. coli* cells with the resulting plasmid library. When transformed, bacteria express green fluorescent protein (GFP) or red fluorescent protein (RFP) if a library insert encodes a promoter on the top or bottom DNA strand, respectively. To map the fluorescence of each bacterium to its respective plasmid insert, we used Sort-Seq (*8*, *13*, *14*, *36*) to separate bacteria with a cell sorter (BD Biosciences, FACSAria III) into eight fluorescence bins corresponding to none, weak, moderate, and strong GFP or RFP expression (Fig. 1B). We then bulk sequenced the plasmid’s DNA inserts from each bin and calculated fluorescence scores in arbitrary units (a.u.) for each insert based on read counts. These scores range from 1.0 a.u. (no expression) to 4.0 a.u. (highest expression; see Materials and Methods).

From the random library, we screened 17,129 unique 150-bp sequences with an average AT content of ~57 ± 6%. We defined a sequence as a promoter if it drove fluorescence expression of at least 1.5 a.u. At this threshold ~13% of sequences with an AT content of 45 to 55% function as promoters on either strand (Fig. 1C), a percentage that aligns with previous studies reporting ~5% (*23*), ~10% (*15*), and ~10 to 20% (*17*) of sequences in similar random DNA libraries encoding promoters on a single strand. With this threshold, 33% (5694) of the 17,129 random sequences encode a promoter, with 27% (4649) driving expression from the top strand, 4% (684) from the bottom strand, and 2% (361) from both strands (fig. S1E).

From the genomic library, we screened 91,866 unique sequences that collectively cover the genome ~2.7-fold (fig. S1B) (*37*). These sequences are ~130 ± 23 bp in length (fig. S1A), with an average AT content of ~47 ± 5%. We note that these sequences were integrated into the plasmid in either genetic orientation, such that a sequence from the top strand of the genome may be located on the top or bottom strand of the plasmid, and vice versa (Materials and Methods and fig. S11). Overall, 32% (29,116) of the 91,866 genomic sequences encode a promoter. More specifically, 25% (23,012) of sequences drive expression from the top strand, 5% (4394) from the bottom strand, and 2% (1710) from both strands (fig. S1E).

Previous reports estimate that ~25% of *E. coli* promoters are constitutively active (*38*). Given that the *E. coli* genome harbors 4639 protein-coding genes and that our sequences provide ~2.7-fold genome coverage, we would thus expect to find of the order of 3131 promoters (4639 × 2.7 × 0.25) in the genomic library. Instead, we find 29,116 promoters or ~9.3× more than expected. Approximately 98.6% (28,696 of 29,116) of these promoters lie in intragenic protein-coding sequences. The average coding sequence therefore contains ~2.3 intragenic promoters each (28,696 intragenic promoters/4639 coding sequences/2.7-fold coverage = 2.3 promoters).

The likelihood that DNA encodes a promoter increases with AT content (*23*), which differs between our two libraries (fig. S1F). To control for AT content when comparing the incidence of promoters, we binned all random and genomic sequences into one of the six intervals that span 5% AT content each. We then calculated the proportion of promoters for all sequences in each bin. Figure 1C reveals that, for both random and genomic libraries, the proportion of promoters increases with AT content. Notably, the proportion of promoters is greater in genomic sequences for all bins. Genomic sequences are ~1.3 times more likely to encode a promoter (average, ~19.0 versus ~14.6%). Thus, there are more promoters in the genome than expected by random chance.

This excess of promoter sequences might be expected for intergenic (nonprotein coding) regions, most of which harbor promoters that are the product of natural selection for gene regulation [see table S1 for matched promoters from RegulonDB; (*39*)]. We thus asked whether the excess only occurs in such regions and is absent from intragenic (coding) regions. To this end, we computationally isolated 964 intergenic DNA sequences mapping exclusively to noncoding DNA from the genomic library and 80,622 intragenic sequences mapping exclusively to coding DNA. We found that DNA originating from both noncoding or coding DNA is still more likely to encode promoters than random DNA (fig. S2, A and B), although the difference is greater for noncoding DNA (~25 versus ~15%). In other words, protein-coding DNA is also more likely to encode a promoter than random DNA (~19 versus ~15%, ~1.3 times more likely). The intragenic promoters that we found occur more often on the antisense strands of these genes (fig. S2C), which is consistent with previous reports about intragenic promoters (*11*, *40*). Because there are more promoters inside coding sequences than expected by random chance, it is possible that some of these intragenic promoters are products of natural selection.

To exclude the possibility that the differences that we observed between random and genomic DNA are caused by variations in sequence length, we subsampled genomic sequences that are exactly 150 bp long, just like the sequences in the random library. These 150-bp genomic sequences are ~1.5 times (23 versus 15%, similar to ~1.3 times) more likely to encode a promoter than random sequences (figs. S1E and S2D). Additionally, the correlation between sequence length and expression level in the genomic library is very small [Pearson’s correlation coefficient (*r*) = 0.01, *P* = 4.91 × 10^−6^; fig. S1C], suggesting that length is not biasing our observations. See fig. S2 for additional analyses.

We also reanalyzed the data under two alternative assumptions, namely, (i) that the number of GFP promoters and RFP promoters in the random library is identical and (ii) that exactly 10% of random DNA sequences have promoter activity on both the GFP and RFP strands (see fig. S3A) (*15*). With this alternative classification strategy, the likelihood of being a promoter still increases with AT content (fig. S3B), and, when accounting for AT content, genomic DNA is still more likely to encode a promoter than random DNA (fig. S3B). There are also ~6.6× more promoters in the genomic library than expected (20,821 observed versus 3131 expected promoters), with ~98% of promoters (20,458 of 20,821) being in intragenic regions. With this alternative strategy, the average coding sequence contains ~1.6 intragenic promoters (20,458 intragenic promoters/4639 coding sequences/2.7-fold coverage).

Together, these analyses demonstrate that the genome’s enrichment of promoters does not just stem from canonical intergenic promoters but also from intragenic sequences, particularly those driving antisense transcription. We estimate that each coding sequence contains ~1.6 to 2.3 intragenic promoters. Because intragenic promoters occur more often than expected by random chance, it is possible that they serve an underappreciated role in transcription. For example, such promoters may induce RNA polymerase collisions to regulate gene expression (*41*).

### The genomic library contains fewer predicted activating and repressing sites than the random library

We next asked whether enrichment of particular TF and σ factor binding sites in our libraries could explain why genomic DNA is more likely to encode a promoter than random DNA. To this end, we obtained a nonexhaustive set of 102 position weight matrices (PWMs) for 93 TFs and 9 σ factors (see Materials and Methods).

Note that a PWM is a matrix derived from protein-DNA binding experiments that represents the frequency of each nucleotide at every position within a TF’s binding site. A PWM has an information content, which reflects the spectrum of DNA sequences that a TF can bind. If a PWM has high information content, then the corresponding TF binds few sequences and is thus highly specific. A PWM can be used to derive a predicted protein binding strength from a query sequence. If this strength is greater than a particular threshold, then we call the query sequence a predicted site for a TF or σ factor (Materials and Methods).

For each PWM, we determined the percentage of sequences in each library that contain at least one predicted site. Not unexpectedly, sites with low information content occur more frequently in random and genomic DNA (Fig. 1D). This is because such sites are expected to occur more often by chance alone. For the genomic library, we expected to find an enrichment of particular predicted TF sites and σ factor sites, because the genome is ~1.3 times more likely to encode a promoter. Unexpectedly, we found that the predicted site frequencies are, on average, ~10% lower in genomic DNA than in random DNA (~16 versus ~26%, paired *t* test, *P* = 7.48 × 10^−12^, see fig. S1G for details). Because TFs can either activate or repress transcription (*42*, *43*), but σ factors exclusively activate transcription (*14*), the most likely explanation for this finding is that genomic DNA may contain fewer binding sites that specifically repress transcription—for brevity, it is less “repressed”—than random DNA, leading to more promoter activity overall.

### De novo promoters emerge more readily from transcriptionally inactive random DNA than from inactive genomic DNA

**Fig. 2.**
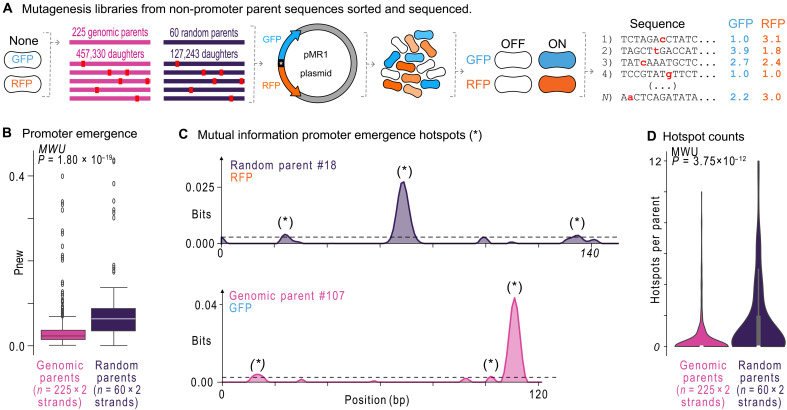
Promoters emerge more readily from random DNA than from genomic DNA. (**A**) We used fluorescence-activated cell sorting (FACS) to isolate sequences in the random and genomic libraries without promoter activity. From these parent sequences, we created mutagenesis libraries of daughter sequences, pooled the parents and their daughters, cloned them into the pMR1 plasmid, and transformed *E. coli* cells with the resulting plasmid libraries. We used Sort-Seq to partition the *E. coli* cells into fluorescence bins and sequenced the DNA inserts from each fluorescence bin to calculate sequence-specific fluorescence scores (see Materials and Methods). (**B**) For each parent and its respective daughters, we calculated *P*_new_, the proportion of daughters with a fluorescence score ≥ 1.5 a.u. We plotted *P*_new_ for the random versus the genomic parents [two-tailed Mann-Whitney *U* (MWU) test, *P* = 1.80 × 10^−19^]. The center line shows the median, the box the interquartile range, and whiskers span ±1 SD. (**C**) We calculated at each nucleotide position *i* along a sequence the mutual information *I*_i_(*b*,*j*) between nucleotide identity (*j* = A, T, C, or G), and fluorescence scores (*f* = 1.0 to 4.0 a.u.) in bits. The *x* axis shows the position *i*, and the *y* axis the sum of mutual information for each nucleotide *j* at this position. The dashed line indicates the 0.0025 bit threshold above which we call a mutual information peak a hotspot (asterisk). Top: The mutual information for random parent #18 and the propensity of mutations to create promoter activity in the bottom (RFP) strand of this parent. Bottom: Mutual information for genome parent #107 and the propensity of mutations to create promoter activity in the top (GFP) strand. (**D**) Hotspot number in each parent in the random and genomic mutagenesis libraries [two-tailed Mann Whitney *U* (MWU) test, *P* = 3.75 × 10^−12^]. See Source Data.

Because the genome encodes more promoters than expected by random chance, we hypothesized that mutations are also more likely to cause promoter activity in genomic DNA than in random DNA. To test this hypothesis, we first used fluorescence-activated cell sorting (FACS) to randomly sample DNA sequences from both libraries that drive neither GFP nor RFP expression (Materials and Methods). Specifically, we isolated 225 such sequences from the genomic library and 60 such sequences from the random library. We call these sequences random and genomic parent sequences, respectively. They do not have promoter activity. Second, for each parent sequence, we created a mutagenesis library of mutant daughter sequences using an error-prone polymerase chain reaction (EP-PCR; Materials and Methods and Fig. 2A). We cloned each of the resulting 285 (60 + 225) mutagenesis libraries into the expression plasmid pMR1 and quantified the potentially new promoter activity driven by each daughter sequence using Sort-Seq (Materials and Methods). We normalized the fluorescence expression level of each daughter to the interval of 1.0 and 4.0 a.u. (highest expression) (Materials and Methods). Overall, the random mutagenesis libraries contain a total of ~1.3 × 10^5^ mutant daughter sequences, i.e., on average, 2121 mutant daughters for each of the 60 parents. The genomic mutagenesis libraries contain ~4.6 × 10^5^ mutant daughter sequences, on average, 2033 mutant daughters for each of the 225 parents (see fig. S4 for library details).

To understand the likelihood of each parent sequence acquiring new promoter activity, we first quantified the probability *P*_new_ that a nonpromoter parent sequence can acquire de novo promoter activity by dividing, for each parent, the number of daughter sequences that drive new gene expression (fluorescence ≥1.5 a.u.) by the total number of daughters (*13*, *14*). For example, random parent #8 has 1487 mutant daughters, of which 144 mutants cause new promoter activity on the top (GFP-coding) strand. Thus, *P*_new_ for this parent equals ~0.0968 (144 of 1487). In other words, 9.68% of random mutations in this parent create a new promoter.

We calculated *P*_new_ for all of the random and genomic parents. Unexpectedly, *P*_new_ is not lower but actually ~3 times higher for random parents than for genomic parents (median *P*_new_ = 0.092 versus 0.029, two-tailed MWU test, *P* = 1.80 × 10^−19^) (Fig. 2B). This result invalidates our hypothesis: New promoters actually emerge more readily from random DNA than from genomic DNA.

We confirmed that the difference in *P*_new_ is significant regardless of the number of point mutations in the daughters (fig. S5A), the number of daughter sequences (fig. S5E), and the length of the parent sequence (fig. S5F). *P*_new_ does not significantly increase with AT content for the random parents (Pearson correlation, *P* = 0.241) and increases only weakly with AT content for the genomic parents (Pearson correlation, *r* = 0.172, *P* = 2.48 × 10^−4^) (fig. S5C). Random parents are still more likely to create new promoters compared to genomic parents when grouped by their AT contents (fig. S5D). A likely reason why promoter activity does not noticeably increase with AT content is that increasing AT content also increases the incidence of binding sites for repressors like H-NS (Histone-like nucleoid-structuring protein), which bind to AT-rich sequences (*44*).

For individual parent sequences, *P*_new_ can be especially high when the parent’s fluorescence score is close to the “promoter” versus “nonpromoter” threshold (1.5 a.u.). In this condition, mutations with small effects can suffice to push the sequence across the classification threshold (*14*). To confirm that differences in basal parental promoter activity are not confounding our conclusion, we computationally identified random and genomic parents whose fluorescence scores equals 1.0 a.u. (rounding to tenths), which corresponds to the majority of parent sequences (fig. S5G). Even for this fluorescence-controlled subset of parents, random parents still have significantly higher values of *P*_new_ than genomic parents. Thus, the basal promoter activity of the parents is unlikely to be confounding our conclusions.

Last, to assess whether the selected parents are statistically representative of genomic and random DNA without promoter activity, we quantified the incidence of DNA hexamers (NNNNNN) in the libraries. We chose hexamers because key promoter elements and such as -10 and -35 boxes, and TF sites are at least 6 bp in length. Specifically, we compared hexamer counts between sequences in (i) the entire genomic library and nonpromoter sequences in this library (fig. S6A) and (ii) nonpromoter genomic sequences and the 225 genomic parents (fig. S6B). No hexamer sequences are enriched in nonpromoter genomic sequences relative to our genomic parents (fig. S6B; Fisher’s exact test), suggesting that the genomic parents are representative of the broader pool of nonpromoter genomic sequences. The same holds for nonpromoter sequences from the random library and our 60 chosen random parents (fig. S6D). We also found that nonpromoter sequences from both the genomic and random library are impoverished in hexamers matching the -10 box (TATAAT / ATTATA; fig. S6, A and C), which is consistent with their lack of promoter activity. Ultimately, these analyses demonstrate that promoter activity (rather than broader sequence composition bias) is the primary distinguishing feature between promoter and nonpromoter sequence classes in our dataset and that our parental sequences are representative of other nonpromoter sequences.

In sum, new promoter activity emerges ~3 times more readily from transcriptionally inactive random DNA than inactive genomic DNA. Because the genome is the product of natural selection and random DNA is not, this finding suggests that the genome may have evolved to prevent spontaneous promoter emergence.

### Transcriptionally inactive genomic DNA harbors fewer emergence hotspots than its random equivalent

We previously demonstrated that mutations preferentially cause new promoters to emerge from “hotspot” regions in a nonpromoter sequence (*13*, *14*). To identify such hotspots in each of our parents, we calculated for each nucleotide position *i* along the parent sequence, the mutual information [*I*_i_(*b*,*j*)] between nucleotide identity (*j* = A, T, C, or G), and fluorescence expression (*f* = 1.0 to 4.0 a.u.) (Materials and Methods) (*8*). This calculation creates a profile of mutual information peaks and valleys for each parent sequence, depicting where promoters can – and cannot – emerge (Fig. 2C) (*7*–*10*, *12*–*14*). We define a hotspot as a mutual information peak greater than 0.0025 bits (Materials and Methods). See fig. S7 for mutual information plots for all parents with at least one hotspot.

The average random parent has ~3.8 times more hotspots than the average genomic parent (~1.5 versus ~0.4 hotspots; two-tailed MWU test, *P* = 3.75 × 10^−12^; Fig. 2D). In other words, genomic parents have fewer regions from which new promoters can emerge. Modifying our definitions of a hotspot does not change this conclusion (fig. S8). We also asked whether the number of hotspots can help to explain why *P*_new_ is significantly lower for genomic parents than for random parents (see Fig. 2B). To this end, we studied the relationship between the number of hotspots and the probability of promoter emergence *P*_new_. We found a strong, positive correlation between the number of hotspots and *P*_new_ (Pearson’s *r* = 0.750 and 0.717, *P* = 5.84 × 10^−23^ and 1.82 × 10^−72^ for random and genomic parents, respectively; fig. S5B). Furthermore, random and genomic parents with the same number of hotspots have virtually the same value of *P*_new_ (fig. S5B). This suggests that the number of unique locations where a promoter can emerge can explain differences in *P*_new_.

In sum, point mutations can create de novo promoters in both transcriptionally inactive random and genomic DNA, but they are about three times more likely to do so in random DNA. The reason is that inactive random DNA harbors more unique regions where new promoters can emerge than inactive genomic DNA (Fig. 2). This finding could be explained by natural selection.

### The *E. coli* genome is impoverished in proto-sites for activating TFs

**Fig. 3.**
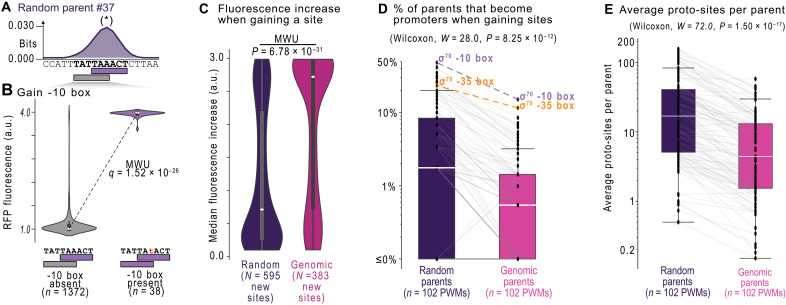
Promoters emerge less frequently in the genome because it harbors fewer proto-binding sites. (**A**) Top: Mutual information *I*_i_(*b*,*j*) (vertical axis) at each position *i* (horizontal axis) between nucleotide identity (*j* = A, T, C or G) and fluorescence (*f* = 1.0 to 4.0 a.u.) in information theoretical units (bits) for random parent *#37*. Bottom: Outlined rectangles and bold sequences correspond to PWM-predicted putative sites for the σ^70^ -10 box (purple) and a region of interest (gray). (**B**) The fluorescence scores of daughter sequences with or without a -10 box in the region of interest from (A). We test the null hypothesis that there is no difference in fluorescence between the two groups [two-tailed Mann-Whitney *U* (MWU) test] and display corrected *q* values using the Benjamini-Hochberg procedure (*58*) (two-tailed MWU test, *q* = 1.52 × 10^−26^). (**C**) The median fluorescence increase when gaining an activating site creates promoter activity (two-tailed MWU test, *P* = 6.78 × 10^−31^). (**D**) The percentage of parent sequences that become promoters when gaining 1 of the 102 different activating sites. Each data point corresponds to a unique PWM. Gray lines pair each PWM between the random and genomic parents. We test the null hypothesis that there is no difference between the median percentages, using a Wilcoxon signed-rank test (*W* = 28, *P* = 8.25 × 10^−12^). (**E**) Analogous to (D) but for the average number of proto-sites per parent instead of the percentage of parent sequences (Wilcoxon signed-rank test, *W* = 72, *P* = 1.50 × 10^−17^). See Source Data.

Mutual information hotspots exist where DNA sequences can either easily gain new sites for activating TFs and σ factors or lose repressing TF sites upon mutation (*13*, *14*). We first focused on activating sites and asked how random and genomic parents gain new TF sites to create new promoters. To this end, we used a nonexhaustive set of 9 σ factor and 93 TF sites (102 PWMs total) and computationally identified daughters, in which the gain of a predicted site by mutation caused a significant increase in gene expression of ≥0.1 a.u., using a two-tailed Mann-Whitney *U* test (Materials and Methods). This procedure predicts, for example, that random parent #37 gains de novo promoter activity when mutations create a binding site (-10 box) for the canonical housekeeping σ^70^ factor (Fig. 3, A and B) (MWU test, *q* = 1.52 × 10^−26^). We call these newly created predicted sites associated with increasing transcriptional activity activating sites. See fig. S9 for additional examples.

Our computational analysis revealed 595 and 383 activating sites in the random and genomic parents, respectively, or ~9.9 new sites per random parent (595 sites per 60 parents) versus ~1.7 per genomic parent (383 sites per 225 parents). The increase in gene expression from gaining new sites was, on average, ~1.7 times higher (+1.97 versus +1.16 a.u.) in the genomic parents compared to the random parents (Fig. 3C) (two-tailed MWU test, *P* = 9.26 × 10^−31^). Simply put, genomic parents are less likely to become promoters by gaining activating sites, but, when they do, they become stronger promoters than random parents.

To understand how each of the predicted sites for 102 TFs and σ factors contributes to de novo promoter emergence, we stratified our analysis by these factors, and calculated the percentage of random and genomic parents that become promoters when gaining a site for each factor. Overall, these percentages are lower in the genomic parents than in the random parents (Fig. 3D) (Wilcoxon signed-rank test, *P* = 8.25 × 10^−12^). For example, ~48% of random parents (29 of 60) acquire promoter activity by gaining a predicted σ^70^ -10 box, while approximately three times fewer genomic parents do (~15%, 34 of 225). Similarly, ~23% of random parents (14 of 60) acquire promoter activity by gaining a σ^70^ -35 box, while almost two times fewer genomic parents do (~12%, 26 of 225). See Source Data for data on the remaining factors.

One possible explanation for this difference is that the genomic parents contain fewer proto-sites, i.e., sequences that resemble TF and σ sites that are ~1 point mutation away from becoming a PWM-predicted site. To test this hypothesis, we searched the parent sequences for DNA sequences yielding a positive PWM score, but where that score falls below the classification threshold as a predicted site (Materials and Methods). Relative to random parents, genomic parents contain almost four times (median, ~16.9 versus ~4.4) fewer proto-sites for our TFs and σ factors (Fig. 3E; Wilcoxon signed-rank test, *P* = 1.50 × 10^−17^). See Source Data for data on all 102 PWMs.

In sum, our analysis shows that emergence hotspots correspond to regions where point mutations create computationally predicted σ or TF binding sites that activate transcription. This observation can explain why promoters emerge less frequently in genomic parents: Transcriptionally inactive genomic DNA contains fewer proto-sites than inactive random DNA. We next turn to hotspots in which point mutations destroy predicted sites that repress transcription.

### Genomic DNA is derepressed compared to random DNA

A paucity of proto-sites in the genomic parents cannot explain why new promoters are stronger when they emerge from genomic parents versus when they emerge from random parents (see Fig. 3C). A potential explanation for this finding goes back to our previous observation that genomic DNA is relatively derepressed compared to random DNA (see Fig. 1D). To test whether the genomic parents are relatively derepressed compared to the random parents, we screened daughter sequences for instances where the loss of a PWM-predicted site for one of the 93 TFs on either DNA strand leads to increasing fluorescence (Materials and Methods). For example, destroying a predicted site for the TF IclR in random parent #30 increases fluorescence by +1.41 a.u. (two-tailed MWU test, *q* = 3.27 × 10^−8^) (see fig. S10B for this and additional examples). We call computationally predicted sites whose destruction is experimentally associated with increased transcription, repressing sites. Like for activating sites, we emphasize that repressing sites are not merely PWM predictions. Their losses are significantly associated with increasing transcription, and they overlap mutual information hotspots (see fig. S10 for examples).

**Fig. 4.**
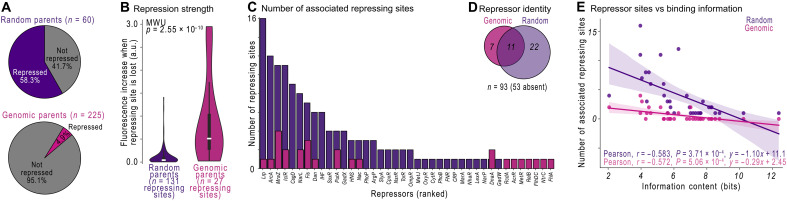
Genomic DNA is bound by fewer putative repressors, but repression is stronger. (**A**) A repressing site in a parent sequence is a site in which mutations that destroy a computationally predicted site result in increased fluorescence (Materials and Methods). We plot the percent of parents with a repressing site for the random (top) and the genomic parents (bottom). (**B**) The median fluorescence increase in a.u. when a repressing site is destroyed in a random versus a genomic parent [two-tailed Mann-Whitney *U* (MWU) test, *P* = 2.55 × 10^−10^]. (**C**) The number of putative repressor sites for random (purple) and genomic (magenta) parents. (**D**) Venn diagram indicating how many of the 93 tested TFs are predicted to bind to the random and genomic parents. (**E**) The number of repressing sites (vertical axis) versus their respective information content (horizontal axis, bits) for the random and genomic parents. The solid line shows a best-fit linear regression line (shaded area, 95% confidence interval). See Source Data.

We identified 131 repressing sites in random parents. They occur in more than half of the random parents (~58%, 35 of 60). Conversely, genomic parents harbor almost five times fewer repressing sites (27 sites versus 131 sites, in only 11 of the 225 genomic parents; Fig. 4A). Parents with a repressing site also have a significantly higher value of *P*_new_ compared to those without a repressing site (two-tailed MWU test, *P* = 3.92 × 10^−4^ and 5.77 × 10^−8^ for random and genomic parents, respectively; see fig. S10K). A likely reason is that there are many ways for a mutation to “break” a repressing site, as opposed to mutations creating new activating sites.

Destroying any 1 of the 27 repressing sites in a genomic parent increases fluorescence, on average, almost 13 times more than destroying a repressing site in a random parent (Fig. 4B; random median, +0.04 a.u.; genome median, +0.51 a.u.; two-tailed MWU test, *P* = 2.55 × 10^−10^). A possible reason is that random parents are repressed by ~2.2 repressing sites each (131 sites per 60 parents), and destroying one sites may not alleviate repression by the remaining sites. Conversely, genomic parents are repressed by only ~0.12 repressing sites each (27 sites per 225 parents), such that a destroyed repressing site is likely to be the only repressing site in a parent. Another potential explanation could be that the strong repression caused by single repressing sites in genomic parents is a product of natural selection.

Fifty-three of the 93 TFs that may be transcriptional repressors are not predicted to significantly repress transcription in the random or genome parents (Fig. 4, C and D; see fig. S10 for examples). For the remaining 40 predicted repressors, 22 repress only the random parents, 7 repress only the genome parents, and 11 repress both the random and genome parents. In other words, the random parents are likely repressed by more unique TFs than the genomic parents (33 versus 18 repressors).

The average repressing site in the genomic parents lies ~28 bp downstream of a PWM-predicted -10 box. In the random parents, repressing sites are ~7 bp upstream of a PWM-predicted -10 box, mapping them to the so-called “spacer” region of the core promoter (fig. S10L). Such arrangements of sites within and around core promoters are frequently observed in putative promoter sequences (*7*).

To summarize, transcriptionally inactive genomic sequences are ~5 times less likely to be subject to repression. However, if they are repressed, their repression is ~13 times stronger than expected by random chance. The lack of repressing sites in genomic DNA suggests that natural selection has removed extraneous repressing sites from the genome.

### Information theory can explain the greater derepression of inactive genomic DNA

We hypothesized that the enrichment of repressing sites in random parents relative to genomic parents is caused by chance alone and by a low DNA binding specificity of the respective repressors. To test this hypothesis, we compared repressor binding specificity (i.e., information content) with the number of repressing sites in both genomic and random libraries. Not unexpectedly, repressors with information-poor binding sites have more such sites, in both random and genomic DNA (Fig. 4E; Pearson correlations; random: *r* = −0.583, *P* = 3.71 × 10^−4^; genome: *r* = −0.572, *P* = 5.06 × 10^−4^; see also Fig. 1D). However, the slope *m* of the line of best fit is almost four times steeper for the random parents and nearly flat in the genomic parents (*m* = −1.10 versus −0.29). This means that the dependency between the number of sites and information content is much weaker for the genomic parents than for the random parents. In other words, random chance can explain the abundance of repressing sites in the random parents, but not in the genomic parents. This abundance requires alternative explanations, such as natural selection.

## DISCUSSION

To summarize our findings, we first compared the incidence of promoters in random, synthetic DNA and in genomic, natural DNA (see Fig. 1). We found that genomic DNA encodes ~1.3 times (~19.0 versus ~14.6%) more promoters than random DNA when we account for differences in AT content. Genomic DNA also contains, on average, ~10% fewer (~16 versus ~26%) computationally predicted TF and σ factor binding sites than random DNA. Previous studies have made similar observations for σ^70^ sites (*15*, *17*). We then compared how de novo promoters emerge from transcriptionally inactive random and genomic “parent” sequences upon mutation (see Fig. 2). We found that promoters are ~3 times (median *P*_new_ = 0.092 versus 0.029) more likely to emerge from inactive random DNA than from inactive genomic DNA. The reason is that these random sequences encode ~3.8 times more emergence hotspots (~1.5 versus ~0.4 hotspots).

Intragenic sequences are ~1.3 times more likely to encode a promoter compared to random DNA. This finding suggests that at least a subset of intragenic promoters is a product of natural selection. Such arguments have been made for σ^28^ promoters, which are primarily found in intragenic regions (*45*). Because most of our intragenic promoters lie on the antisense strand (see fig. S2C), it is possible that they induce head-on RNA polymerase collisions to regulate gene expression (*41*). Alternatively, constraints on nucleotide and codon usage imposed by the space of possible protein-coding sequences may bias coding sequences toward being (but not becoming) prokaryotic promoters, although it has been shown that prokaryotic genomes exhibit codon biases against serendipitously encoding promoter motifs (*11*, *30*).

The paucity of emergence hotspots in nonpromoter genomic DNA has two explanations. First, such DNA encodes fewer proto-sites (see Fig. 3) that are easily converted into a promoter by mutation (~16.9 versus ~4.4 proto-sites per parent). This scarcity lowers the likelihood that mutations create new activating sites, leads to ~3.8 times fewer emergence hotspots, and ultimately makes promoters ~3 times less likely to emerge by mutations in inactive genomic DNA. Second, the majority (~58%) of random parents are weakly repressed (see Fig. 4), compared to a minority (~5%) of genomic parents that are strongly repressed. This relative lack of repression in the genomic parents may also help to explain why intragenic DNA is ~1.3 times more likely to be a promoter than random DNA.

The scarcity of predicted activating and repressing sites in transcriptionally inactive regions of the genome may be a product of natural selection. For example, selection may remove promoter-like sequences and TF binding sites to prevent the spontaneous emergence of promoters (*27*–*29*). It may also affect binding sites indirectly by acting on other traits associated with gene expression (*46*). For example, it may reduce conflicts between transcription and replication during cell division (*47*), or it may help lowly expressed factors sequester to single binding sites in the genome (*48*–*50*). To disentangle these different roles of natural selection remains an exciting task for future work.

Regardless of the selective pressures involved, the scarcity of activating proto-sites and repressing sites in the genome will influence the evolution of new promoters. First, a paucity of repression biases the genome toward producing strong de novo promoters (see Fig. 3C). We speculate that this could lead to larger changes in fitness upon emergence, which would result in selection (rather than genetic drift) acting on their activity (*26*). Second, because the genome harbors few activating proto-sites, it is biased against creating de novo promoters.

Emergent promoters can create new transcripts for de novo genes (*21*, *22*) or transcribe previously inactive genes (*18*–*20*). Such new transcripts can potentially confer a selective advantage. We show here that the *E. coli* genome biases the likelihood that promoters emerge, where they emerge, how they emerge (by gaining or losing binding sites), which TFs they use, and how strongly they drive gene expression. By extension, such biases will influence the emergence of evolutionary adaptations and innovations.

## MATERIALS AND METHODS

### Plasmid MR1

Plasmid MR1 (pMR1) is derived from the pRV2 plasmid by replacing the kanamycin resistance cassette with a chloramphenicol (cm) resistance gene (*35*). It carries the BBa_J34801 ribosomal binding site (AAAGAGGAGAAA), positioned 6 bp upstream of the GFP (Leu-Val-Ala, LVA) start codon. On the opposite strand, a predicted ribosomal binding site (AAGGGAGG) is located 5 bp upstream of the mCherry start codon. The plasmid contains the p15A origin of replication, which confers a low-to-medium copy number (*35*). A map of the 4009-bp plasmid is available in the associated GitHub repository: https://github.com/tfuqua95/random-genomic.

### Position weight matrices

To generate PWMs, we acquired lists of putative binding sites for TFs from RegulonDB (*39*) and individual publications for σ factors (*51*–*54*). Using the motifs package from Biopython (v1.79) (*55*), we created PWMs for these factors, assuming a uniform background frequency of 25% for each nucleotide. We then classified any one query sequence as a binding site if its respective PWM score (in bits) was greater than or equal to the natural logarithm of the PWM’s information content (in nats). This threshold is also referred to as the Patser threshold (*56*). In Fig. 3E, we also classified sequences as proto-binding sites if the PWM score was greater than 0.00 bits, but less than this Patser threshold.

### Creating the wild-type genomic library

See fig. S11 for an overview of how we created the wild-type genomic library. To isolate genomic DNA, we inoculated a culture of DH5α electrocompetent *E. coli* cells (Takara, Japan, product no. 9027) and allowed the cells to grow overnight with shaking at 230 rpm at 37°C. The following day, we extracted the genomic DNA using a Wizard kit (Promega, USA, product no. A1120) according to the manufacturer’s instructions, eluting the final product in 50 μl of water (fig. S11A). We estimated the genomic DNA concentration using a NanoDrop One spectrophotometer (Thermo Fisher Scientific, USA) as ~10 ng in a total volume of 50 μl. We sheared the genomic DNA into fragments using a focused ultrasonicator (Covaris E220), with the following parameters: (i) duty factor, 10%; (ii) peak incident power, 175 W; (iii) cycles per burst, 200; and (iv) time, 430 s, as described on the manufacturer’s website (www.covaris.com/e220-focused-ultrasonicator-500239)

We separated the fragmented genomic DNA using agarose gel electrophoresis with a 3% agarose gel and manually excised DNA between 100 and 200 bp in length. We purified this DNA using a QIAGEN QIAquick Gel Purification Kit (QIAGEN, The Netherlands, product no. 28706) according to the manufacturer’s instructions. To repair any DNA damage caused by sonication, we used a NEBNext repair kit [New England Biolabs (NEB), USA, product no. M6630] to create blunt ends. We did this by adding 270 ng of the sheared DNA (18 μl), 2.5 μl of the provided buffer, 1.25 μl of the provided enzymes, and 3.25 μl of water (total volume of 25 μl). We incubated this reaction for 30 min at 20°C in a C1000 Touch Thermal Cycler (Bio-Rad, USA) with the thermal cycler lid temperature at 30°C. We purified the reaction product using a QIAquick PCR Purification Kit (QIAGEN, The Netherlands, product no. 28104) according to the manufacturer’s instructions.

We next ligated an adenine (A) base to the 3′ ends of each fragment using the NEBNext dA-Tailing Module (NEB, USA, product no. E6053) (fig. S11B) by combining 20 μl of blunt-end DNA (21 ng/μl), 2.5 μl of buffer, 1.5 μl of the Klenow fragment, and 1.0 μl of H_2_O (total volume of 25 μl) and incubating the reaction at 37°C for 35 min in a C1000 Touch Thermal Cycler with the lid heated to 45°C. We purified this product with a QIAquick PCR Purification Kit (QIAGEN, The Netherlands, product no. 28104) according to the manufacturer’s instructions.

To create upstream and downstream double-stranded DNA sequences homologous to our expression vector, we synthesized and annealed two sets of complementary oligonucleotides (IDT, Coralville, USA). For each double-stranded DNA, one of the oligonucleotides included a 5′ phosphorylation modification for downstream ligation (see data S7 for primer sequences for oligo1-4). We annealed complementary oligonucleotides by incubating them in an equimolar ratio at 95°C for 2 min in a thermocycler (C1000 Touch Thermal Cycler, Bio-Rad, USA) and subsequently decreasing the temperature by increments of 5°C every 90 s, until a final temperature of 25°C had been reached.

To ligate the double-stranded DNA upstream and downstream of the adenylated genomic DNA, we used a T4 ligase master mix (NEB, USA, product no. M0202), with 3 μl of 10× T4 buffer, 1 μl of the upstream annealed adaptor (100 μM), 1 μl of the downstream annealed adaptor (100 μM), 20 μl of the adenylated genomic DNA (16 ng/μl, 320 ng total), 1.5 μl of T4 Ligase, and 3.5 μl of H_2_O. We incubated this reaction mix at room temperature for 2 hours and heat-inactivated the reaction at 65°C for 10 min. Following the ligation, we excised the product using agarose (1%) gel electrophoresis and purified the isolated DNA using a QIAquick Gel Purification Kit (QIAGEN, The Netherlands, product no. 28706) according to the manufacturer’s instructions.

We performed a polymerase chain reaction (PCR) to amplify the ligated products using Q5 high-fidelity polymerase (NEB, USA, product no. M0491) according to the manufacturer’s instructions and with primers “pMR1_insert_forward” and “pMR1_insert_reverse” provided in data S7. We incubated the reaction for 30 cycles (annealing during each cycle at 55°C for 30 s and extending primers at 72°C for 30 s) using a thermal cycler (C1000 Touch Thermal Cycler, Bio-Rad, USA). We then separated the PCR products on a 1% agarose gel, excised the product between 200 and 500 bp, and purified the gel product using a QIAquick Gel Purification Kit (QIAGEN, The Netherlands, product no. 28706). We cloned the genomic library into the pMR1 using Gibson Assembly (fig. S11C) and transformed the products into DH5α cells as described in the “Molecular cloning and transformations” section.

### Creating the wild-type random library

To create the wild-type random library, we synthesized a 150-bp N-mer sequence with flanking sequences homologous to our reporter vector (pMR1) (IDT, Coralville, USA). See data S7 for the homologous DNA sequences. Using Q5 high-fidelity polymerase (NEB, USA, product no. M0491), we amplified the fragment according to the manufacturer’s instructions and with the primers shown in data S7. We incubated the PCR reaction for 30 cycles (annealing in each cycle at 55°C for 30 s and extending the primers at 72°C for 30 s) using a thermal cycler (C1000 Touch Thermal Cycler, Bio-Rad, USA). We then separated the PCR products on a 1% agarose gel, excised DNA of ~200 to 300 bp with a scalpel, and purified the gel product using a QIAquick Gel Purification Kit (QIAGEN, The Netherlands, product no. 28706) according to the manufacturer’s instructions. We cloned the library into the pMR1 plasmid using Gibson Assembly and transformed the resulting plasmid library into DH5α cells as described in the “Molecular cloning and transformations” section.

### Molecular cloning and transformations

To clone inserts into the pMR1 dual reporter plasmid (*35*), we used Q5 polymerase (NEB, USA, product no. M0491) to make linearized copies of the pMR1 plasmid flanking the Bam HI (GGATCC) and Eco RI (GAATTC) cut sites. To this end, we used primers “pMR1_vector_forward” and “pMR1_vector_reverse” listed in data S7 and the following reagents: 20 μl of Q5 buffer, 2 μl of dNTPs (deoxynucleotide triphosphates, Thermo Fisher Scientific, USA, product no. R0191), 10 μM forward primer, 10 μM reverse primer, 1 μl of the insert, 1 μl of Q5 polymerase, and 71 μl of H_2_O. We performed the cloning reaction in a thermal cycler (C1000 Touch Thermal Cycler, Bio-Rad, USA) for 30 cycles of annealing at 55°C for 30 s and extending at 72°C for 2 min and 30 s. We separated the cloning products on a 1% agarose gel and extracted the DNA band of interest using a scalpel. To purify the gel product, we used a QIAquick Gel Purification Kit (QIAGEN, The Netherlands, product no. 28706) according to the manufacturer’s instructions and eluting the DNA in 50 μl of H_2_O.

We then used NEBuilder (Gibson Assembly) (NEB, USA, product no. E2621) to clone inserts into the linearized plasmid pMR1. To this end, we added 100 ng of pMR1, 5 μl of the NEBuilder mastermix, 24 ng of insert, and H_2_O to a total volume of 10 μl. We incubated this cloning reaction for 1 hour at 50°C. Subsequently, we transformed 2 μl of the cloned product into 100 μl of DH5α electrocompetent cells (Takara, Japan, product no. 9027) using an electroporator (Bio-Rad, USA, MicroPulser). Following electroporation, we allowed the cells to recover in 1 ml of Super Optimal Broth with Catabolite Repression Medium (SOC; Merck, product no. CMR0002) for 1.5 hours, incubating at 37°C and shaking at 230 rpm. We plated 5 μl of the transformed culture onto an LB agar plate supplemented with cm (100 μg/ml). We added 2 ml of LB-cm to the remaining 995 μl of transformed product and cultured the cells overnight.

The following morning, we counted the number of colonies on the agar plate to estimate the total number of unique inserts in the library. We then stored 1 ml of the liquid library culture with 667 μl of 60% glycerol at −80°C for later use. With the remaining 2 ml of the liquid culture, we isolated the library using a QIAprep Spin Miniprep Kit (QIAGEN, Germany, product no. 27104).

### Defining fluorescence bins for Sort-Seq

We defined the boundaries for G1-G4, R1-R4, GFP-on/off, and RFP-on/off bins using the fluorescence readouts of three control reporter plasmids (fig. S12). The first was a negative control consisting of the pMR1 plasmid backbone without an insert. The second was a positive control for green fluorescence, which contains the *bba_J23110* promoter inserted into pMR1 oriented toward the GFP coding sequence. The third was a positive control for red fluorescence, which contains the *bba_J23110* promoter inserted into pMR1 oriented toward the RFP coding sequence.

For the green fluorescence bins, we first defined the lower boundary of G1 (no green fluorescence) as the lowest measured green fluorescence in the negative control (pMR1 with no insert) and the lowest measured green fluorescence for the RFP-positive control. We use a lower bound to avoid counting and isolating debris such as salts and cell waste. To define the upper boundary of G1, we used the highest measured green fluorescence in the negative control, and the highest measured green fluorescence in the RFP-positive control. To define the boundaries of R1 (no red fluorescence), we repeated this procedure but switched the positive RFP control with the GFP-positive control.

To define the lower boundaries of G4 and R4 (strong green and red fluorescence, respectively), we used the mean fluorescence of the respective (green or red) positive control. We did not define an upper bound for bins G4 and R4. We note that the GFP distribution for the GFP-positive control was bimodal, where the lower (left) peak likely corresponds to cells not harboring the insert (see fig. S12B). For this reason, we take the mean fluorescence of the second (right) peak to define the lower bound of G4 (fig. S12B).

To define the boundaries of G2 and G3, we divided the lower boundary of G4 and the upper boundary of G1 in half, such that the lower bound of G3 becomes the upper bound of G2. Similar for R2 and R3, we divided the lower boundary of R4 and the upper boundary of R1 in half, such that the lower bound of R3 becomes the upper bound of R2. See fig. S12 for an overview of the controls and bin boundaries. See also the Sort-Seq (wild-type random and genomic libraries)” and “Sort-Seq (mutagenesis libraries)” sections for sorting details.

For the genome mutagenesis library, we sorted cells into the four bins GFP-ON, GFP-OFF, RFP-ON, and RFP-OFF, as opposed to the eight bins just described (G1-G4 and R1-R4). GFP-OFF is analogous to G1 and RFP-OFF is analogous to R1. The lower bound of GFP-ON is analogous to the lower bound of G2 and does not have an upper bound. Likewise, for RFP-ON, the lower bound is analogous to the lower bound of R3 and does not have an upper bound. See fig. S12 and the “Sort-Seq (mutagenesis libraries)” section for sorting details.

### Sort-Seq (wild-type random and genomic libraries)

We inoculated 100 μl of the random library or genomic library into separate cultures of 10 ml of LB supplemented with cm (100 μg/ml) and incubated the two cultures at 37°C overnight with shaking at 230 rpm. The following morning (~16 hours later), we washed and resuspended the bacteria with phosphate-buffered saline (PBS). We then sorted the random and genomic libraries independently into four green fluorescence bins (G1, G2, G3, and G4) and four red fluorescence bins (R1, R2, R3, and R4), using a cell sorter (BD Biosciences, FACSAria III) (see the “Defining fluorescence bins for Sort-Seq” section for binning procedure) as follows: For the wild-type random library (day 1), we sorted 1,440,000 cells into G1, 180,000 cells into G2, 120,000 cells into G3, 60,000 cells into G4, 4,320,000 cells into R1, 540,000 cells into R2, 360,000 cells into R3, and 180,000 cells R4. For the wild-type genomic library (day 1), we sorted 1,200,000 cells into G1, 150,000 cells into G2, 100,000 cells into G3, 864 cells into G4, 1,200,000 cells into R1, 150,000 cells into R2, 33,647 cells into R3, and 17,101 cells into R4.

After sorting (8 bins for the two libraries, 16 bins in total), we added 1 ml of SOC (Merck, product no. CMR0002) to each culture and incubated the cultures at 37°C (with shaking at 230 rpm) for 2 hours to help the bacteria recover. We then supplemented each culture with an additional 2 ml of LB and 0.3 μg of cm (total volume of 3 ml). We incubated the eight cultures for both libraries overnight at 37°C with shaking at 230 rpm. The following morning (~16 hours later), we repeated the sorting and recovery procedure, this time, resorting each culture into its corresponding bin as the day before, in triplicates, as follows: For the wild-type random library (day 2), we resorted 1,080,000 total cells into G1 [divided into three equal-volume replicates (r1, r2, and r3), ~360,000 cells per replicate], 104,222 total cells into G2, 25,678 total cells into G3, 45,000 total cells into G4, 1,080,000 total cells into R1, 135,000 total cells into R2, 90,000 total cells into R3, and 45,000 total cells into R4 (three replicates, eight bins, 24 final cultures). For the wild-type genomic library (day 2), we resorted 3,600,000 total cells into G1, 450,000 total cells into G2, 300,000 total cells into G3, 30,000 total cells into G4, 3,600,000 total cells into R1, 450,000 total cells into R2, 100,941 total cells into R3, and 51,303 total cells into R4 (three replicates, eight bins, 24 final cultures).

We allowed the bacteria in each of the 24 cultures to recover for 2 hours in SOC before adding an additional 2 ml of LB with 0.3 μg of cm. We then incubated the cultures overnight at 37°C at 230 rpm. The following morning (~16 hours later), we stored 1 ml of each library culture with 667 μl of 60% glycerol at −80°C. With the remaining 2 ml of the culture, we isolated the plasmids from each culture using a QIAprep Spin Miniprep Kit (QIAGEN, Germany, product no. 27104).

### Creating the mutagenesis libraries

We inoculated 100 μl of the random library and the genome library into separate cultures of 10 ml of LB supplemented with cm (100 μg/ml) and incubated the two cultures at 37°C overnight at 230 rpm. The following morning (~16 hours later), we washed and resuspended the bacteria with PBS. Using a cell sorter (BD Biosciences, FACSAria III), we isolated 2,000,000 and 1,000,000 bacteria that collectively fluorescence in both bin G1 (no GFP fluorescence) and bin R1 (no RFP fluorescence) from the random library and the genomic library, respectively (two sorted cultures after sorting). We allowed the sorted bacteria to recover in 1 ml of SOC (Merck, product no. CMR0002) for 2 hours before we added an additional 2 ml of LB and 0.3 μg of cm (to a total volume of 3 ml). We incubated the two cultures overnight at 37°C with shaking at 230 rpm. The following morning (~16 hours later), we repeated the sorting and recovery procedure for these cultures, this time isolating 459,185 bacteria that sort into G1 and R1 from the negative random library culture and 1,611,091 bacteria that sort into G1 and R1 from the negative genomic library culture. We refer to these cultures as the “negative” random (genomic) cultures.

We plated the negative cultures onto LB-Agar petri dishes supplemented with cm (100 μg/ml). We then picked individual colonies using pipette tips, resuspending each colony in 10 μl of H_2_O. We selected 96 colonies from the negative random culture and 396 colonies from the negative genomic cultures. We then carried out individual EP-PCRs for the DNA in each colony. For each EP-PCR, we added 6.9 μl of H2O, 2 μl of 5× GoTaq reaction buffer, 0.2 μl of GoTaq polymerase (Promega, USA, product no. M3001), 0.1 μl of primer pMR1_insert_forward, 0.1 μl of primer pMR1_insert_reverse, 0.2 μl of dNTPs (Thermo Fischer, USA, product no. R0191), and 0.2 μl of MnCl_2_ at 15 mM (for mutagenesis) to a reaction “master-mix.” For each EP-PCR, we added 9 μl of this master-mix to 1 μl of the resuspended colony. We then performed the reactions in a thermal cycler (C1000 Touch Thermal Cycler, Bio-Rad, USA) for 30 cycles of annealing at 55°C for 30 s and extending at 72°C for 30 s. We pooled the products of the random EP-PCR into one test tube and the genomic EP-PCR into another test tube. We separated the pooled reaction products electrophoretically on a 1% agarose gel, and extracted the DNA bands of interest using a scalpel. To purify the gel products, we used a QIAquick Gel Purification Kit (QIAGEN, The Netherlands, product no. 28706) according to the manufacturer’s instructions. We separately cloned and transformed the two mutagenized insertion libraries into pMR1 and DH5α electrocompetent cells as described in the “Molecular cloning and transformations” section.

### Sort-Seq (mutagenesis libraries)

We inoculated 100 μl of the random mutagenesis library and the genomic mutagenesis library into two separate cultures of 10 ml of LB supplemented with cm (100 μg/ml) and incubated the two cultures at 37°C overnight at 230 rpm. The following morning (~16 hours later), we washed and resuspended the bacteria in PBS.

We sorted the genomic mutagenesis library into two green fluorescence bins (GFP-ON and GFP-OFF) and two red fluorescence bins (RFP-ON and RFP-OFF). In addition, we sorted the random mutagenesis library into four green fluorescence bins (G1, G2, G3, and G4) and four red fluorescence bins (R1, R2, R3, and R4) (see the “Defining fluorescence bins for Sort-Seq” section for binning procedure). GFP-ON and GFP-ON are, respectively, analogous to G1 and R1. However, we combined the read counts (postsequencing) from bins R2-R4 and G2-G4 to make these combined bins analogous to RFP-ON and GFP-ON, respectively, as described in the “Processing mutagenesis library sequencing results” section.

For the random mutagenesis library (day 1), we sorted 1,000,000 cells into G1, 70,826 cells into G2, 11,129 cells into G3, 12,576 cells into G4, 1,000,000 cells into R1, 324,742 cells into R2, 52,550 cells into R3, and 24,276 cells into R4. For the genomic mutagenesis library (day 1), we sorted 2,000,000 cells into GFP-OFF, 5754 cells into GFP-ON, 2,000,000 cells into RFP-OFF, and 193,330 cells into RFP-ON.

We allowed the sorted bacteria in each bin to recover in 1 ml of SOC (Merck, product no. CMR0002) for 2 hours before supplementing each culture with an additional 2 ml of LB and 0.3 μg of cm (total volume of 3 ml). We incubated the cultures overnight at 37°C, with shaking at 230 rpm. The following morning (~16 hours later), we repeated the sorting and recovery procedure, this time resorting each culture into its corresponding bin as follows: For the random mutagenesis library (day 2), we resorted 3,000,000 total cells into G1, 200,013 total cells into G2, 250,000 total cells into G3, 375,000 total cells into G4, 3,000,000 total cells into R1, 1,500,000 total cells into R2, 750,000 total cells into R3, and 375,000 total cells into R4 (eight bins, eight cultures, no replicates). For the genomic mutagenesis library (day 2), we resorted 6,000,000 total cells into GFP-OFF, 360,000 total cells into GFP-ON, 6,000,000 total cells into RFP-OFF, and 1,960,687 total cells into RFP-ON (four bins, four cultures, no replicates).

We allowed the sorted cultures to recover for 2 hours in SOC before adding an additional 2 ml of LB with 0.3 μg of cm and incubating the cultures overnight at 37°C, with shaking at 230 rpm. The following morning (~16 hours later), we stored 1 ml of each culture with 667 μl of 60% glycerol at −80°C. With the remaining 2 ml of the culture, we isolated the plasmids from each culture using a QIAprep Spin Miniprep Kit (QIAGEN, Germany, product no. 27104) for Illumina sequencing.

### Illumina sequencing

From the plasmids isolated from each sorted library bin, we used a PCR to amplify the plasmid inserts for next-generation sequencing. Specifically, for each PCR, we mixed 20 μl of Q5 buffer (NEB, USA, product no. 4091), 2 μl of dNTPs (Thermo Fisher Scientific, USA, product no. R0191), 10 μM reverse primer “Illumina_reverse_primer,” 1 μl of the insert, 1 μl of Q5 polymerase, and 71 μl of H_2_O. In addition, we added to each reaction mix a multiplexing forward primer (10 μM) with a unique barcode for each fluorescence bin and replicate. See data S7 for a list of primers and barcodes. We performed the reaction in a thermocycler (C1000 Touch Thermal Cycler, Bio-Rad, USA), with 30 cycles of annealing at 55°C for 30 s and extending at 72°C for 2 min and 30 s. We separated the PCR products on a 1% agarose gel and extracted the DNA band of interest using a scalpel. To purify the gel product, we used a QIAquick Gel Purification Kit (QIAGEN, The Netherlands, product no. 28706) according to the manufacturer’s instructions. We pooled the PCR products and sequenced them using Illumina paired-end read sequencing (Eurofins GmbH, Germany) on an Illumina NovaSeq 6000 (Illumina, USA) with an S4 flow cell and paired-end 150-bp runs.

### Processing wild-type library sequencing results

For the wild-type libraries, we merged the sequencing reads into paired-end reads using Flash2 (*57*). We oriented each paired-end read based on the flanks of the read and categorized the read to its respective bin and replicate number using the unique forward primer index. To map the genomic fragments to their respective genomic coordinates, we used bowtie2 (v2.3.5.1) to create an index map of the *E. coli* genome, and mapped the fragments to these indices using SAMtools (v1.6).

We excluded reads from further analysis, if they (i) were not present at least once in the unsorted library, (ii) were not present in at least four separate bins, (iii) were shorter than 100 bp or longer than 200 bp, and (iv) did not map to the genome (for reads from the genomic library). Data S1 and data S2 are csv files of the remaining (included) reads from the random wild-type library and the genomic wild-type library, respectively, together with their fluorescence scores.

### Processing mutagenesis library sequencing results

For the mutagenesis libraries, we also merged the sequencing reads into paired-end reads using Flash2 (*57*). We oriented each paired-end read based on the flanks of the read and categorized the read by its respective bin and replicate number using the unique forward primer index.

As described in the “Sort-Seq (mutagenesis libraries)” section, we had sorted the random mutagenesis library into eight fluorescence bins (G1-G4 and R1-R4), while we had sorted the genomic mutagenesis library into only four fluorescence bins (GFP-OFF, GFP-ON, RFP-OFF, and RFP-ON). G1 and R1 are analogous to GFP-OFF and RFP-OFF, while the sum of G2, G3, and G4 is analogous to GFP-ON, and the sum of R2, R3, and R4 is analogous to RFP-ON (see the “Defining fluorescence bins for Sort-Seq” section for binning procedure and fig. S12). To compare the results from the two mutagenesis libraries, we thus pooled the reads from G2, G3, and G4 to constitute the corresponding GFP-ON bin. Similarly, we pooled the reads of R2, R3, and R4 to constitute the corresponding RFP-ON bin.

To map the daughter (mutagenized) sequences to their respective parents (wild-type), we partitioned the daughters into groups based on their length in base pairs. For each group of sequences with the same length, we represented the Hamming distance between every sequence pair as a contingency matrix. From the contingency matrix, we identified clusters of sequences with a Hamming distance of no more than 10 mutations. From each cluster, we then identified the parent sequence as the consensus DNA sequence of the cluster.

We excluded a parent and its daughters from the dataset if (i) we could not find the parent sequence in the respective wild-type library, (ii) there were less than 500 daughters per parent, and (iii) the parent and daughter sequences were shorter than 100 bp or longer than 200 bp. The resulting filtered sequence datasets are contained in data S3 for the random mutagenesis library and data S4 for the genomic mutagenesis library.

### Calculating fluorescence scores

We calculated fluorescence scores (${\text{fluor}}_{r}$) for each technical replicate (r1, r2, and r3) and fluorophore (GFP and RFP) using Eq. 1

$${\text{fluor}}_{r}=\frac{\sum_{1}^{n}\left(f\times {\text{Reads}}_{f}\right)}{\sum_{1}^{n}\left({\text{Reads}}_{f}\right)}$$ (1)

When this equation is applied to the wild-type libraries, the index *f* represents integer-encoded fluorescence bins (*f* = 1, 2, 3, and 4 for G1, G2, G3, and G4 or for R1, R2, R3, and R4), and ${\text{Reads}}_{f}$ is the number of reads within each fluorescence bin *f*. For each sequence, we computed the average fluorescence score across replicates using Eq. 2

$$\text{fluor}=\frac{{\text{fluor}}_{r1}+{\text{fluor}}_{r2}+{\text{fluor}}_{r3}}{3}$$ (2)

We do not have replicates for the mutagenesis libraries and therefore do not take the mean of the replicates for the fluorescence scores. Therefore $\text{fluor}={\text{fluor}}_{r}$ for the mutagenesis libraries.

### Mutual information

For each parent sequence, we quantified the mutual information $I_{i}$ between the nucleotide identity b at position i (where 1 ≤ i ≤ the length of the parent sequence) in its corresponding daughter sequences and their associated fluorescence values. We performed this computation using Eq. 3, following the method outlined in (*8*)

$$I_{i}\left(b,f_{\text{round}}\right)=\sum_{b}\sum_{f_{\text{round}}}p_{i}\left(b,f_{\text{round}}\right){\text{log}}_{2}\frac{p_{i}\left(b,f_{\text{round}}\right)}{p_{i}\left(b\right)\times p\left(f_{\text{round}}\right)}$$ (3)

Here, *b* corresponds to all possible nucleotides (b=A,T,C,or G), and $f_{\text{round}}$ corresponds to the daughter fluorescence scores fluor rounded to the nearest integer, yielding four discrete fluorescence levels ($f_{\text{round}}$ = 1, 2, 3, and 4 a.u.) (see the “Processing wild-type library sequencing results” and “Processing mutagenesis library sequencing results” sections for calculating fluorescence scores). In other words, $f_{\text{round}}$ is a discretized version of the continuous fluorescence score fluor defined in the “Processing wild-type library sequencing results” and “Processing mutagenesis library sequencing results” sections.

The quantity $p_{i}\left(b\right)$ denotes the relative frequency of A, T, C, or G at position (*i*) in the daughter sequences; $p\left(f_{\text{round}}\right)$ is the relative frequency of rounded fluorescence scores being equal to 1, 2, 3, and 4 a.u.; and $p_{i}\left(b,f_{\text{round}}\right)$ is the corresponding joint frequency.

We visualize mutual information hotspots, regions of high mutual information, by smoothening mutual information values as a function of position *i* with the scipy package ndimage Gaussian filter (parameter alpha = 2). See mutual information values in Source Data. From these smoothened data, we identified mutual information peaks at each position *i*, such that $I_{i}\left(b,f_{\text{round}}\right)>0.0025$ bits, and $I_{i-1}\left(b,f_{\text{round}}\right)<I_{i}\left(b,f_{\text{round}}\right)>I_{i+1}\left(b,f_{\text{round}}\right)$.

To confirm that our finding of random parents containing more hotspots than genomic parents (see Fig. 2D) is not a by-product of our thresholding and smoothing procedures, we repeated this analysis using three different thresholds (0.000, 0.0025, and 0.005 bits) and four different smoothing alpha parameters (0, 1, 2, and 3) (see fig. S8).

### Identifying significant associations between mutations creating or destroying sites

To identify regions within each parent sequence where binding-site creating (destroying) mutations are associated with increasing fluorescence, we performed the following computational screen for each parent sequence of length *L* (base pairs). We moved a sliding window of length *X*, corresponding to the length (*X*) of the tested PWM along each parent sequence in 1-bp increments (from position 1 to position *L*-*X*). At each position, we scanned all corresponding mutant daughter sequences for the presence or absence of a PWM-predicted binding site, as defined in the “Position weight matrices” section. We then grouped the corresponding fluorescence scores of daughter sequences with the binding site as “positives,” and those lacking the site as “negatives.”

If both the “positive” and “negative” groups contained more than 10 fluorescence scores, we tested whether binding site presence was associated with higher fluorescence using a two-sided Mann-Whitney *U* test (via mannwhitneyu in scipy.stats). We applied this procedure at each window position, on both DNA strands, for all parent sequences, and for both green and red fluorescence measurements. To account for multiple hypothesis testing, we applied the Benjamini-Hochberg procedure (*58*) with a false discovery rate of 0.05 and considered regions with *P* value corrected *q* values below 0.05 (*q* < 0.05) to be significant. Data S5 and data S6 contain the results of this analysis for the random mutagenesis library and the genome mutagenesis library, respectively.

To specifically explore how mutations create binding sites (see Fig. 3), we focused on sites that (i) were gained (not lost), (ii) emerged on the same strand associated with the fluorescence increase, and (iii) had an associated fluorescence increase greater than or equal to 0.1 a.u..

To specifically explore how mutations destroy binding sites (see Fig. 4), we focused on sites that (i) were lost, (ii) occurred on either DNA strand associated with a fluorescence increase, and (iii) showed an associated fluorescence increase greater than 0.0 a.u. We performed these analyses for the 93 binding sites (PWMS) listed in data S5 and data S6. We did not analyze associations where losing a predicted σ factor binding site increases fluorescence, because our previous work demonstrates that such sites are false positives confounded by the creation or destruction of other predicted sites (*14*).
