# Supplementary material for: De novo promoters emerge more readily from random DNA than from genomic DNA
Source: Sci Adv. 2026 May 29;12(22):eaec2554. doi: 10.1126/sciadv.aec2554 (PMC13220856; doi:10.1126/sciadv.aec2554)
Supplement: Supplementary file 1 — Figs. S1 to S12 Legend for table S1 Data S1 to S7 Legend for source data [file sciadv.aec2554_sm.pdf]

Science Advances
AAAS

Supplementary Materials for

De novo promoters emerge more readily from random DNA than from genomic DNA

Timothy Fuqua and Andreas Wagner

Corresponding author: Andreas Wagner, andreas.wagner@uzh.ch

Sci. Adv. **12**, eaec2554 (2026)
DOI: 10.1126/sciadv.aec2554

The PDF file includes:

Figs. S1 to S12
Legend for table S1
Data S1 to S7
Legend for source data

Other Supplementary Material for this manuscript includes the following:

Table S1
Source Data

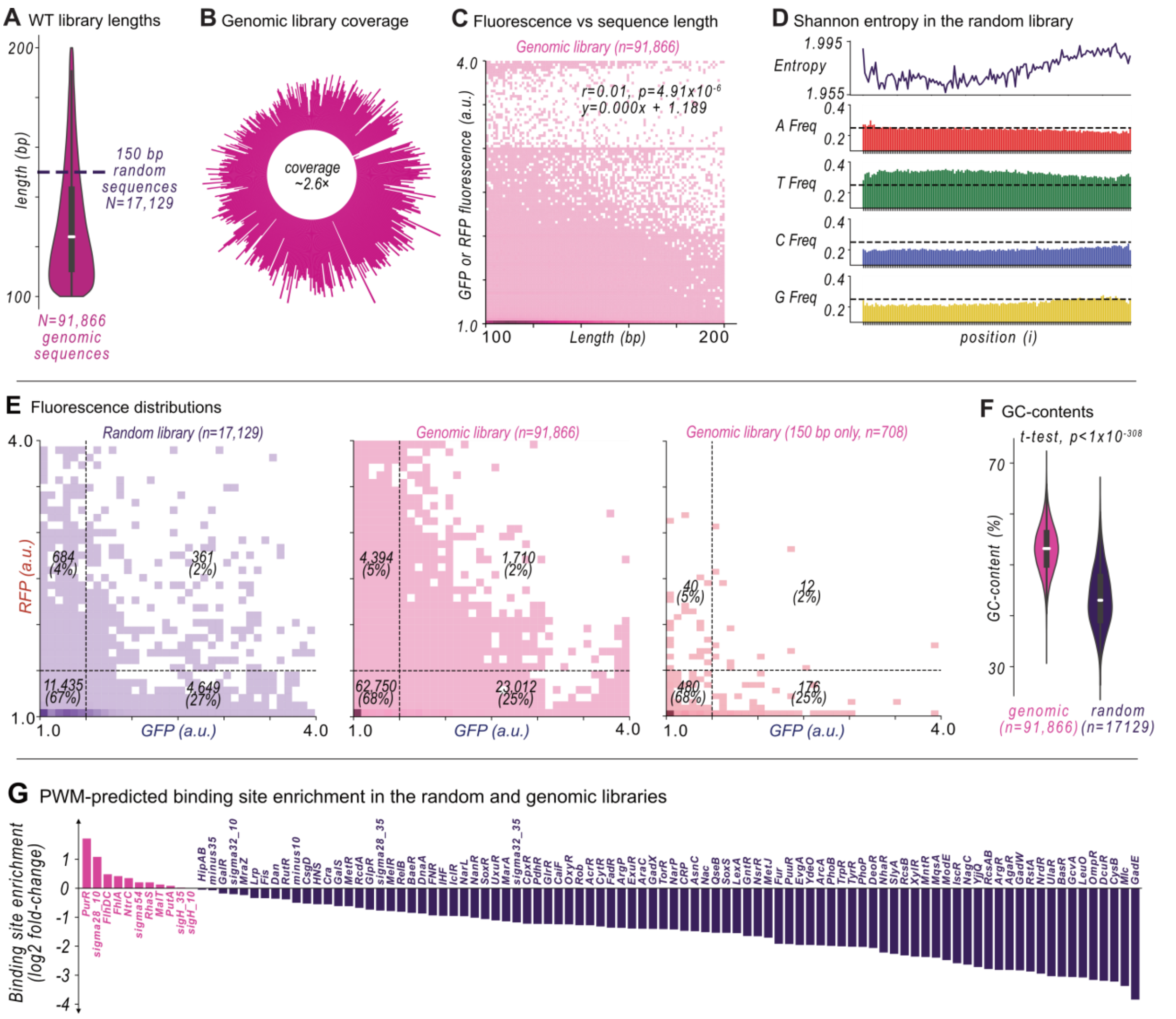

Fig. S1. Wild-type library compositions. (A) The distribution of sequence lengths in the genomic library. The horizontal dashed line corresponds to the fixed 150 bp length of sequences in the random library. The white line shows the median, the gray box the interquartile range (IQR), whiskers span ±1 standard deviation, and the violin area reflects a kernel density estimate of the distribution. **(B)** A histogram of the coordinates of sequences in the genomic library. **(C)** Scatter plot of fluorescence scores (y-axis) vs sequence length (x-axis) of sequences in the genomic library. We tested the null hypothesis that there is no association between fluorescence and length. Although this association is significant, it is very weak (Pearson's r = 0.01, $p=4.91\times10^{-6}$, n=91,866). The fitted linear equation on top of the panel was calculated using the method of least squares (line of best fit not shown). **(D)** Top: the Shannon Entropy of nucleotide identity at each position i in the random library. Bottom four panels: the frequency of each nucleotide (A, T, C, or G) at each position. The dashed horizontal line represents a frequency of 0.25. **(E)** Scatter plot of fluorescence scores (x-axis GFP, y-axis RFP) for sequences in the libraries (left: random library, middle: genomic library, right: genomic library with exactly 150 bp in length. Dashed lines at 1.5 a.u. indicate our threshold for promoter activity. **(F)** GC-content of the genome (magenta, left) vs the random (purple, right) libraries (two-tailed t-test, $p<1\times10^{-308}$). **(G)** For 102 position-weight matrices (PWMs) for transcription factors and sigma (σ) factors we plot the percentage of sequences in each library (purple: random, magenta: genome) that encode at least one putative binding site for each factor (See **Fig 1D**). We show the $\log_2$ fold-enrichment of these frequencies as a bar plot. See **Source Data**.

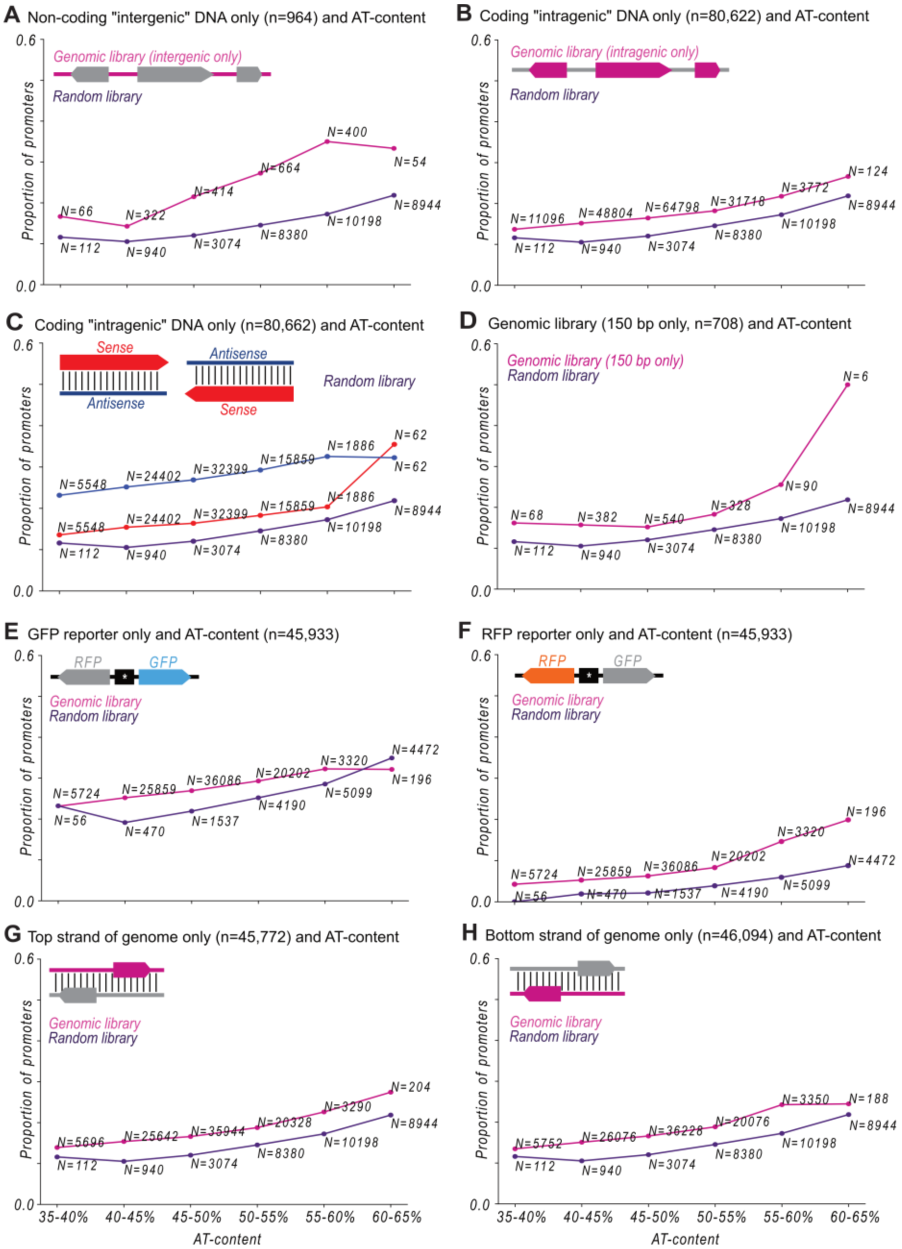

Fig. S2. Proportion of promoters and AT-content. (**A**) The proportion of DNA sequences with promoter activity (fluorescence ≥ 1.5 arbitrary units, a.u.) in random DNA (purple) vs genomic library sequences that lie exclusively in intergenic (non-coding DNA) regions (magenta, n=964 unique sequences). **(B)** Analogous to (A) but comparing random DNA to sequences in the genomic library that lie exclusively in intragenic (inside coding DNA) regions (magenta, n=80,622 unique sequences). **(C)** Analogous to (B) but for the proportion of genomic sequences that lie exclusively inside coding DNA (n=80,622 unique sequences) if they are on the sense-strand (red) or antisense strand (blue). **(D)** Analogous to (A) but comparing random DNA sequences to genomic sequences exclusively 150 bp in length. **(E)** Analogous to (A) but comparing only promoter activity measured from the green fluorescent protein (GFP) fluorescence reporter. **(F)** Analogous to (E) but for the red fluorescent protein (RFP) fluorescence reporter. **(G)** Analogous to (A) but comparing random DNA to genomic sequences that lie on the top strand in their native genomic context. **(H)** Analogous to (G) but for genomic sequences that lie on the bottom strand in their native genomic context. See **Source Data**.

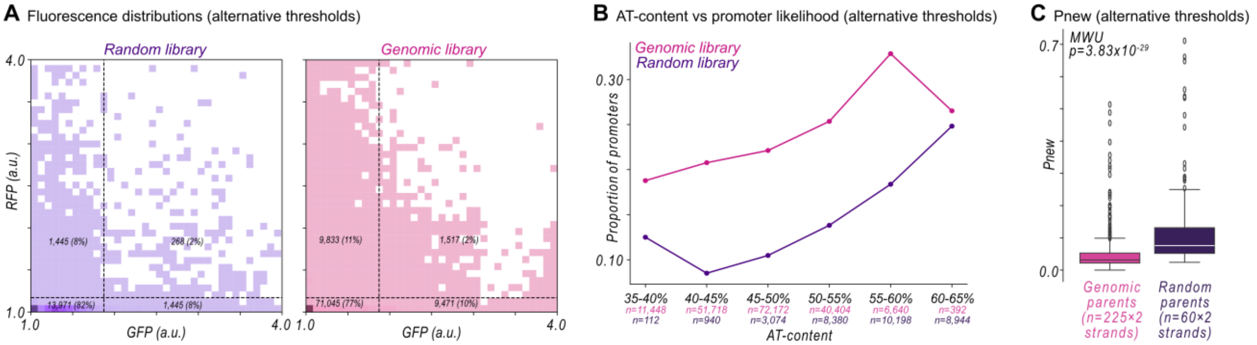

Fig S3. Alternative thresholds for defining promoters lead to similar conclusions. (A) Scatter plot of fluorescence scores (x-axis GFP, y-axis RFP) for sequences in the libraries (left: random library, right: genomic library). Dashed vertical line at 1.872 arbitrary units (a.u.) and 1.170 a.u. correspond to the 90th percentile of GFP and RFP scores, respectively. Sequences with fluorescence scores above these alternative thresholds are considered "promoters" in the subsequent panels. **(B)** The probability that a DNA sequence in the random and genomic libraries is a promoter (y-axis) as a function of its AT-content (x-axis). **(C)** For each parent and its respective daughters, we calculated P_{new}, the proportion of daughters with a fluorescence score ≥ 1.872 a.u. (GFP) or ≥ 1.170 a.u. (RFP). We plotted P_{new} for the random parents and for the genomic parents. P_{new} is significantly greater for random sequences (two-tailed Mann-Whitney U [MWU] test, $p=3.83\times10^{-29}$). The center line shows the median, the box the interquartile range (IQR), and whiskers span ±1 standard deviation. Outliers are shown as outlined circles. See **Source Data**.

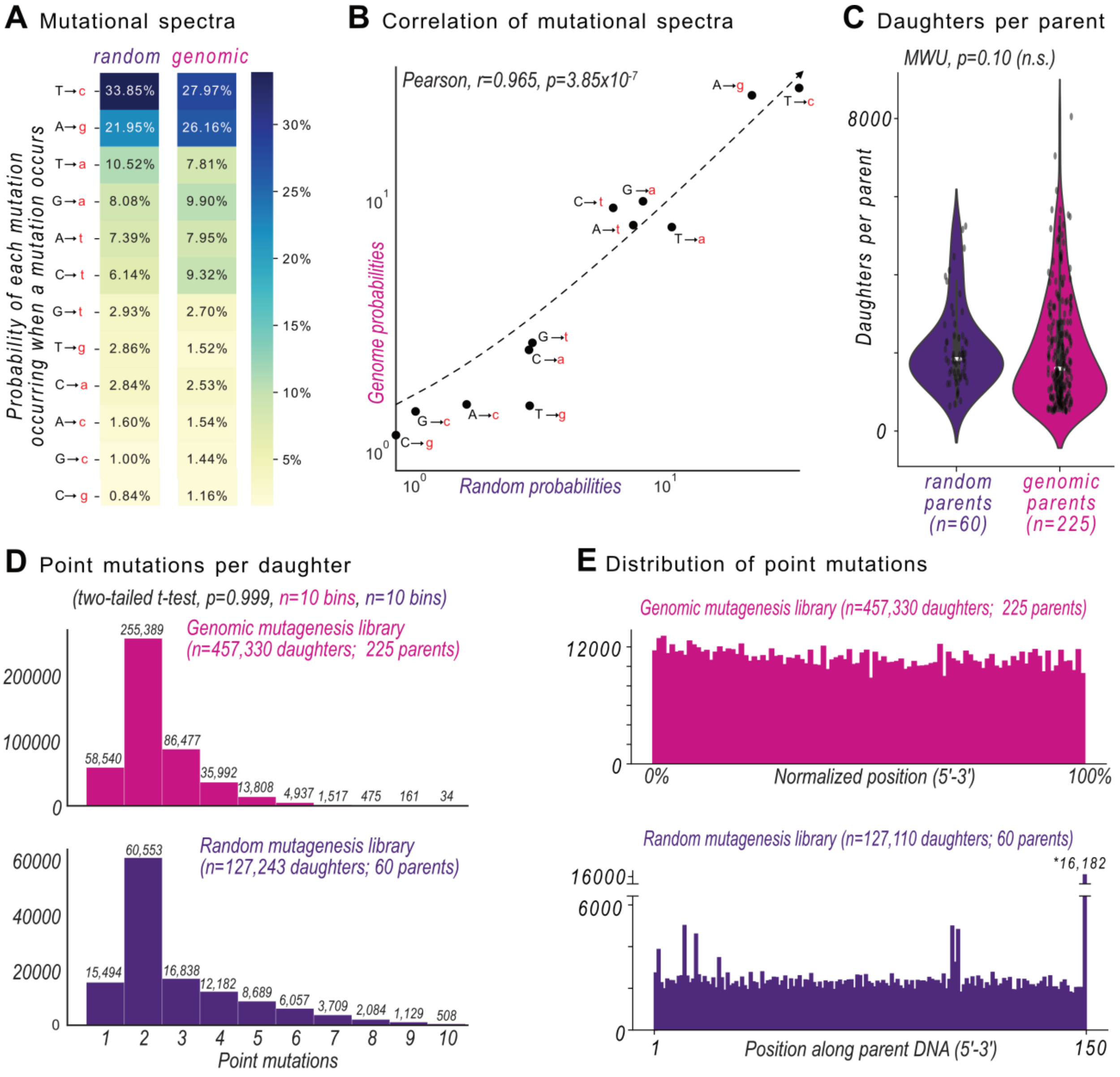

Fig. S4. The mutagenesis libraries. (A) A heatmap depicting the frequency of different point mutations in the daughter sequences. **(B)** A scatter plot comparing the different point mutation frequencies (see A) in the random mutagenesis library and the genomic mutagenesis library. We tested the null hypothesis that the two frequencies are not correlated (Pearson's r=0.965, p=3.85×10^{-7}). The fitted equation (dashed line) was calculated using the method of least squares. **(C)** The number of daughter sequences for each parent in the random library (purple, left) vs the genomic library (magenta, right; two-tailed Mann Whitney U test, p=0.10, n.s.=not significant). **(D)** Distribution of the number of point mutations in each daughter sequence (top: genome mutagenesis library, bottom: random mutagenesis library). **(E)** The cumulative number of point mutations along the parent sequences, normalized to the percentage of sequence length. Top: genome mutagenesis library, bottom: random mutagenesis library. The reasons why the last position of sequences in the random mutagenesis library experienced more mutations are not known. However, mutual information hotspots do not consistently overlap with the final positions of each parent sequence (see **Fig S7**), suggesting it is not affecting the downstream analyses. See **Source Data**.

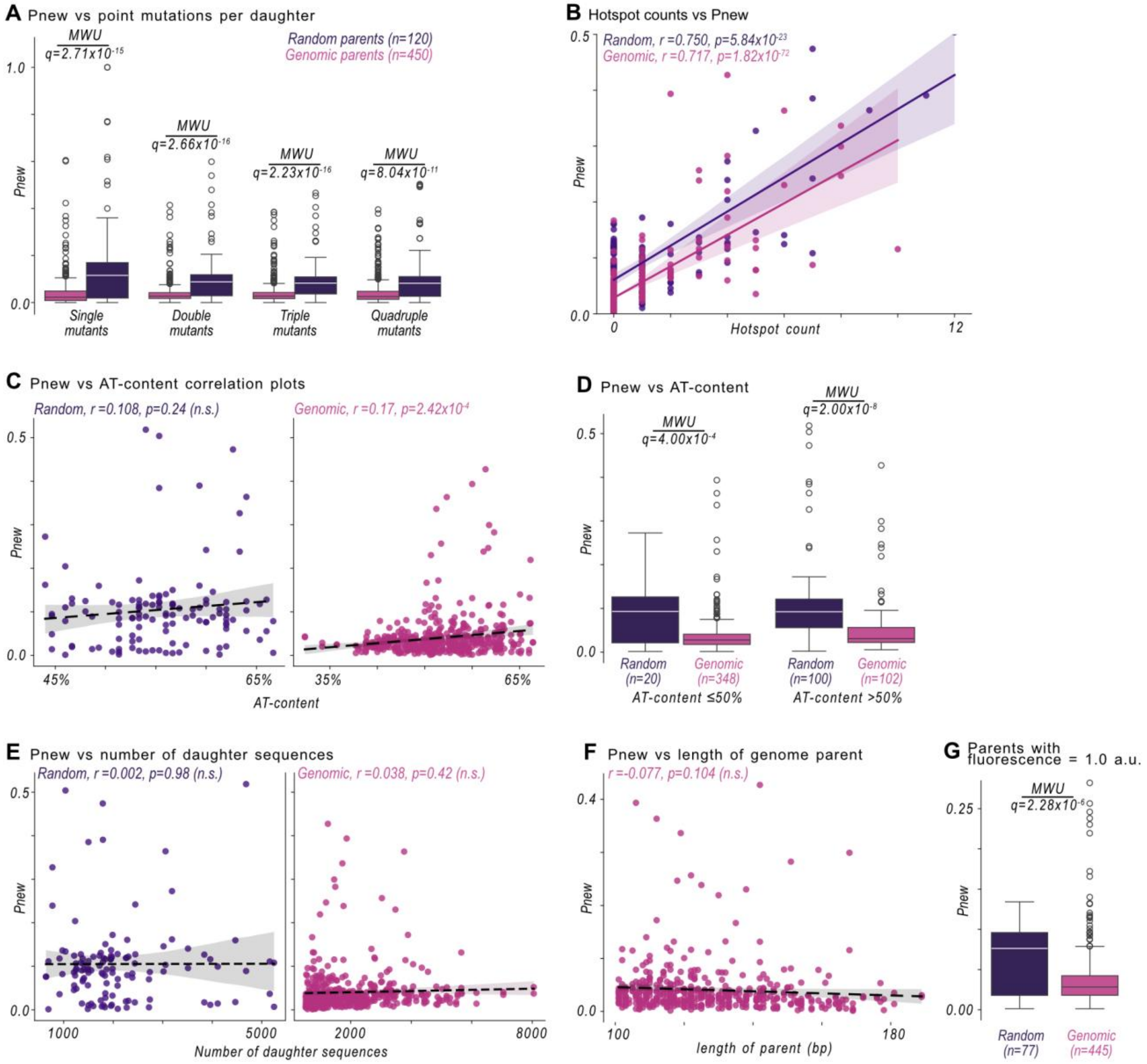

Fig. S5. Mutagenesis libraries and P_{new}. (A) The distribution of P_{new} - the probability that a sequence acquires promoter activity by mutation (fluorescence score ≥1.5 a.u., vertical axis) – vs the number of point mutations (horizontal axis) for parent sequences in the genomic mutagenesis library (left member of each pair, magenta) and the random mutagenesis library (right member of each pair, purple). For each box plot, the central line corresponds to the median, the filled rectangle to the interquartile range (IQR), and the whiskers indicate ±1 standard deviation. Outlined circles indicate outliers. For each boxplot pair, we test the null hypothesis that there is no difference in distributions using a two-tailed Mann-Whitney U (MWU) test. We correct the output p-values to q-values using the Benjamini-Hochberg procedure (*42*). Single mutants: $q=2.71\times10^{-15}$, double mutants: $q=2.66\times10^{-16}$, triple mutants: $q=2.23\times10^{-16}$, quadruple mutants: $q=8.04\times10^{-11}$. Higher numbers of mutations have too small sample sizes for analysis. **(B)** A scatter plot of the number of mutual information hotspots (vertical axis, see also Fig 2) and P_{new} (horizontal axis, see also panel A). The null hypothesis that there is no association between these quantities is rejected (random, purple points: Pearson's r=0.750, $p=5.84\times10^{-23}$; genomic, magenta points: Pearson's r=0.717, $p=1.82\times10^{-72}$). The solid line shows the best-fit linear regression model, with the shaded area indicating the 95%

confidence interval. **(C)** Analogous to (B) but for P_{new} vs AT-content in the random parents (left, purple, r=0.108, p=0.24, n.s. = not significant) and the genomic parents (right, magenta, r=0.17, $p=2.42\times10^{-4}$). **(D)** Analogous to (A) but comparing P_{new} in random (purple) and genomic parents (magenta) for those parents with an AT-content ≤ 50% (left pair, $q=4.00\times10^{-4}$) and > 50% (right pair, $q=2.00\times10^{-8}$). **(E)** Analogous to (C) but for P_{new} vs the number of daughter sequences. **(F)** Analogous to (C) but for P_{new} vs the length of the parent sequence (bp). **(G)** Analogous to (B) but for the P_{new} values of parents with fluorescence scores that equal 1.0 arbitrary units (a.u.). See **Source Data**.

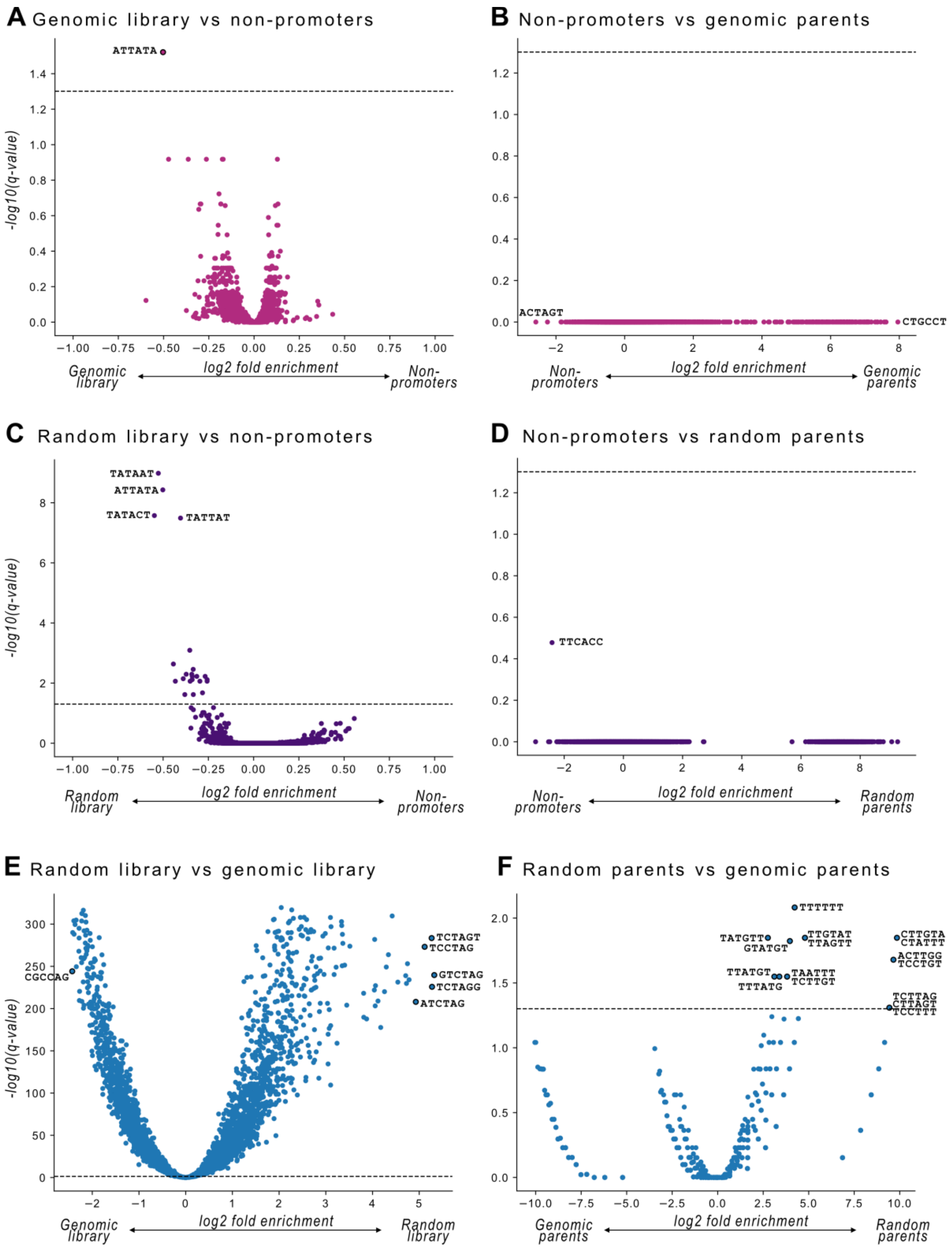

Fig. S6. Library hexamer enrichment. (A) A volcano plot comparing the frequency of hexamers (NNNNNN, 4096 total) between the totality of the *E. coli* genomic library and only its non-promoter sequences (x-axis, log2 fold enrichment). For each hexamer, we test the null hypothesis that there is no difference in its frequencies between the two parts of the library using a Fisher's exact test, and correct for multiple hypothesis testing using the Benjamini-Hochberg procedure (*42*). Corrected q-values are plotted

on the y-axis on a negative log10 scale (dashed horizontal line: 0.05). `ATTATA` is the only significantly under-represented hexamer in non-promoter sequences. It is the reverse complement of the -10 box consensus sequence (`TATAAT`), which is consistent with the fact that this part of the library lacks promoter activity, and highlights the importance of the -10 box. **(B)** Analogous to (A) but comparing all non-promoter sequences from the genomic library to the genomic parents. There is no significant difference in hexamer frequencies between the libraries (Fisher's exact test), suggesting the genomic parents are representative of non-promoter sequences in the *E. coli* genome. **(C)** Analogous to (A) but comparing hexamer incidence between the random library and the non-promoter sequences in this library. Significantly enriched hexamers correspond to instances of the -10 box (`TATAAT, ATTATA, TATACT, TATTAT`), highlighting again the importance of the -10 box for promoter activity. **(D)** Analogous to (A) but comparing non-promoter sequences from the random library with random parents. There is no significant difference in hexamer frequencies, suggesting that the random parents are representative of non-promoter sequences from the random library. **(E)** Analogous to (A) but comparing hexamer incidence between the random library and the genomic library. **(F)** Analogous to (A) but comparing random parents and genomic parents. See **Source Data**.

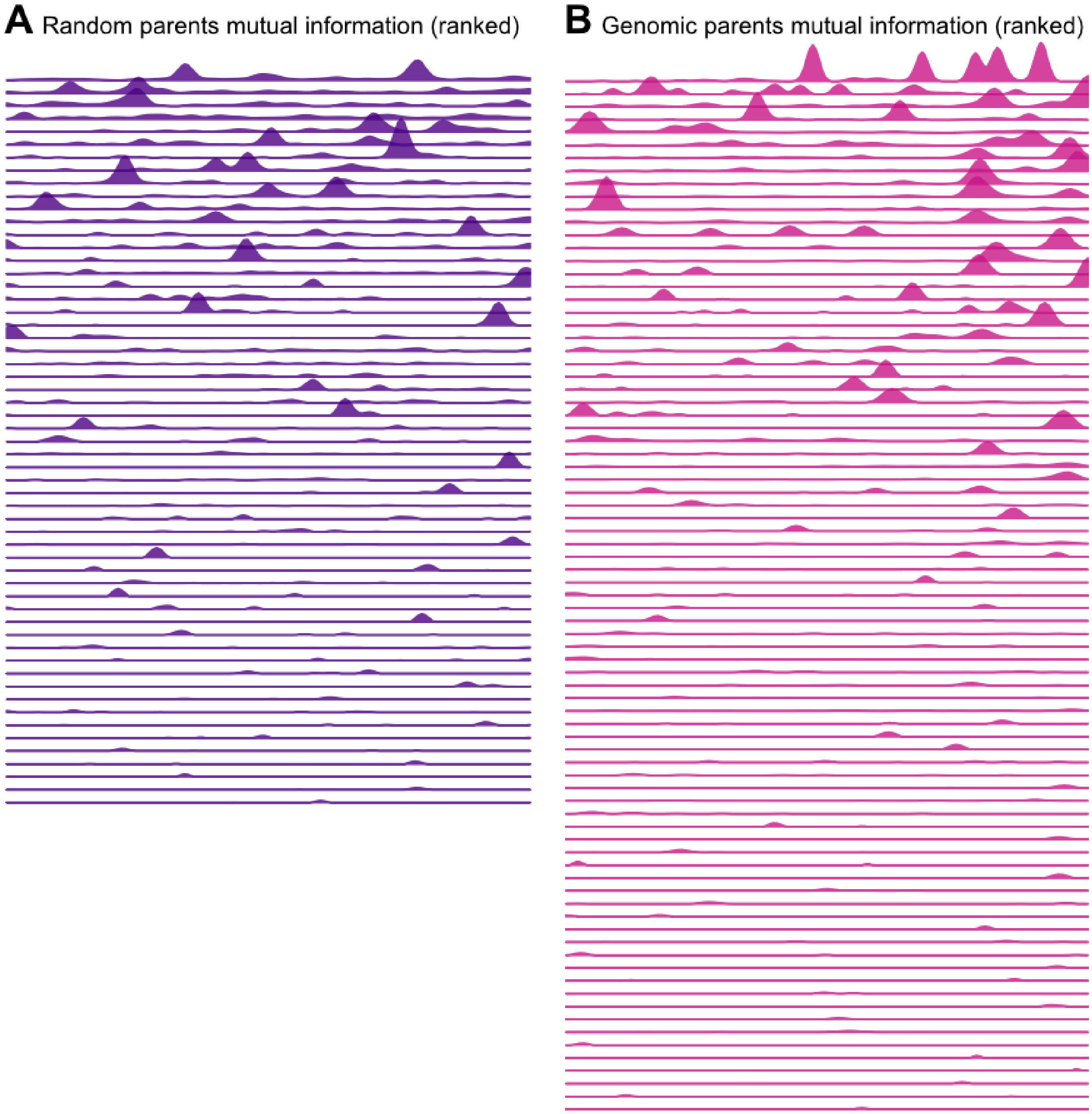

Fig. S7. Mutual information hotspots. (**A,B**) We calculate at each position (i) along a parent sequence (horizontal axes), the mutual information $I_i(b,j)$, vertical axes) between the nucleotide identity *(j = A, T, C or G)*, and the fluorescence score *(f = 1.0 – 4.0 a.u.)* in information theoretical units (bits). Positions are normalized to percentages of parent length, because the genomic parents vary in length. Each plot shows the mutual information of a single parent sequence and fluorophore / genetic orientation. Plots are ranked based on the total sum of the mutual information. Parent sequences without any mutual information hotspots are not shown (see **Methods**). **(A)** Random parents. **(B)** Genomic parents. See **Source Data**.

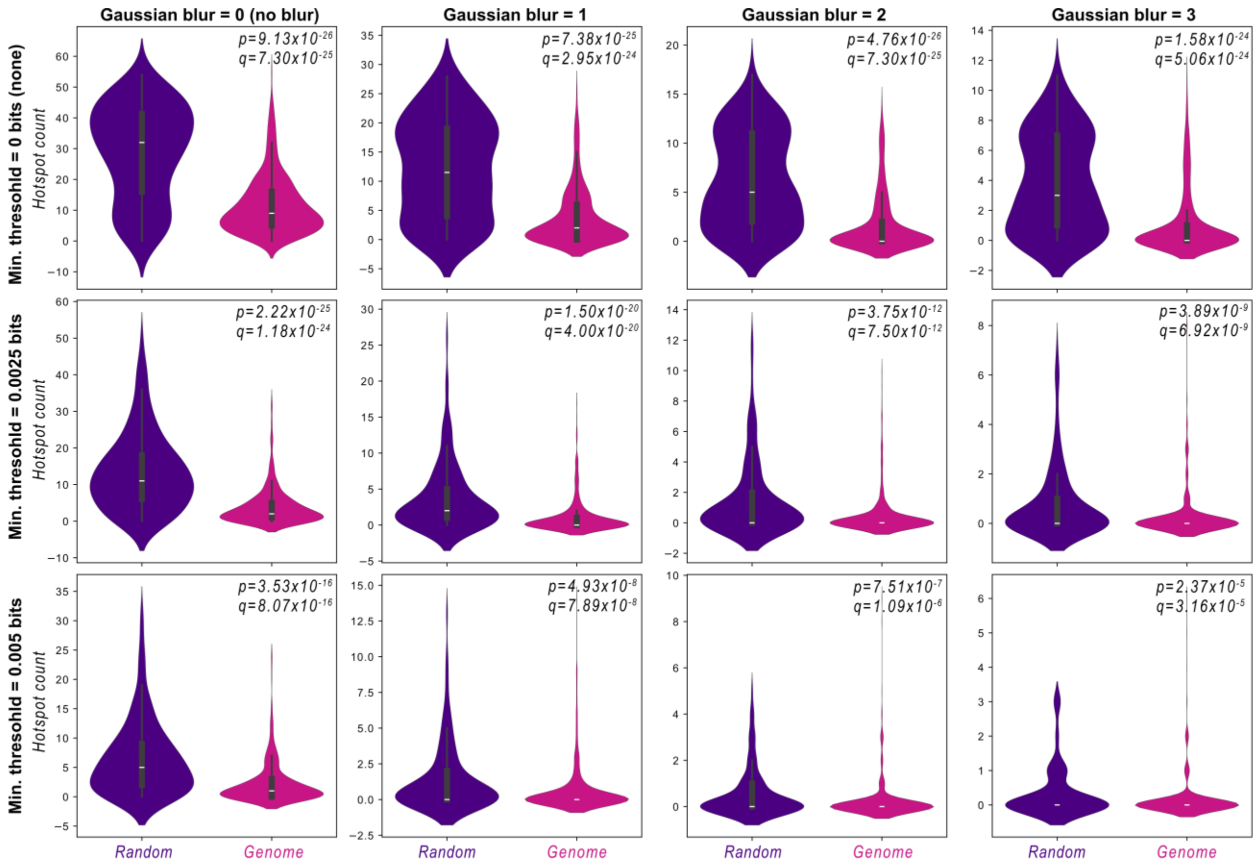

Fig. S8. Gaussian blurring and minimal information thresholding vs the number of hotspots. Each panel shows a pair of violin plots that compares the distribution of the number of mutual information hotspots (see also **Fig 2**) between parents in the random library (purple, left plot) and parents in the genome library (magenta, right plot). The white line shows the median, the gray box the interquartile range (IQR), whiskers indicate ±1 standard deviation, and the filled shape reflects a kernel density estimate. For data in each panel, we test the null hypothesis that the distributions are the same, using a two-tailed Mann-Whitney U test to compute a p-value. We account for multiple-hypothesis testing with the Benjamini-Hochberg procedure (*42*) to compute a corresponding q-value. Not significant (n.s.) values are written in red. (Left to right panels) Before calculating the distribution of hotspots, we apply a Gaussian filter (see **Methods**) using the scipy.ndimage (v1.13.1) function gaussian_filter, incrementally increasing the sigma (σ) parameter for the Gaussian filter from left to right (σ = 0, 1, 2, and 3). (Top to bottom panels) Peaks must be above a minimum threshold to be considered. We incrementally increase this threshold (top to bottom) from 0.000 bits to 0.0025 and 0.005 bits. See **Source Data**.

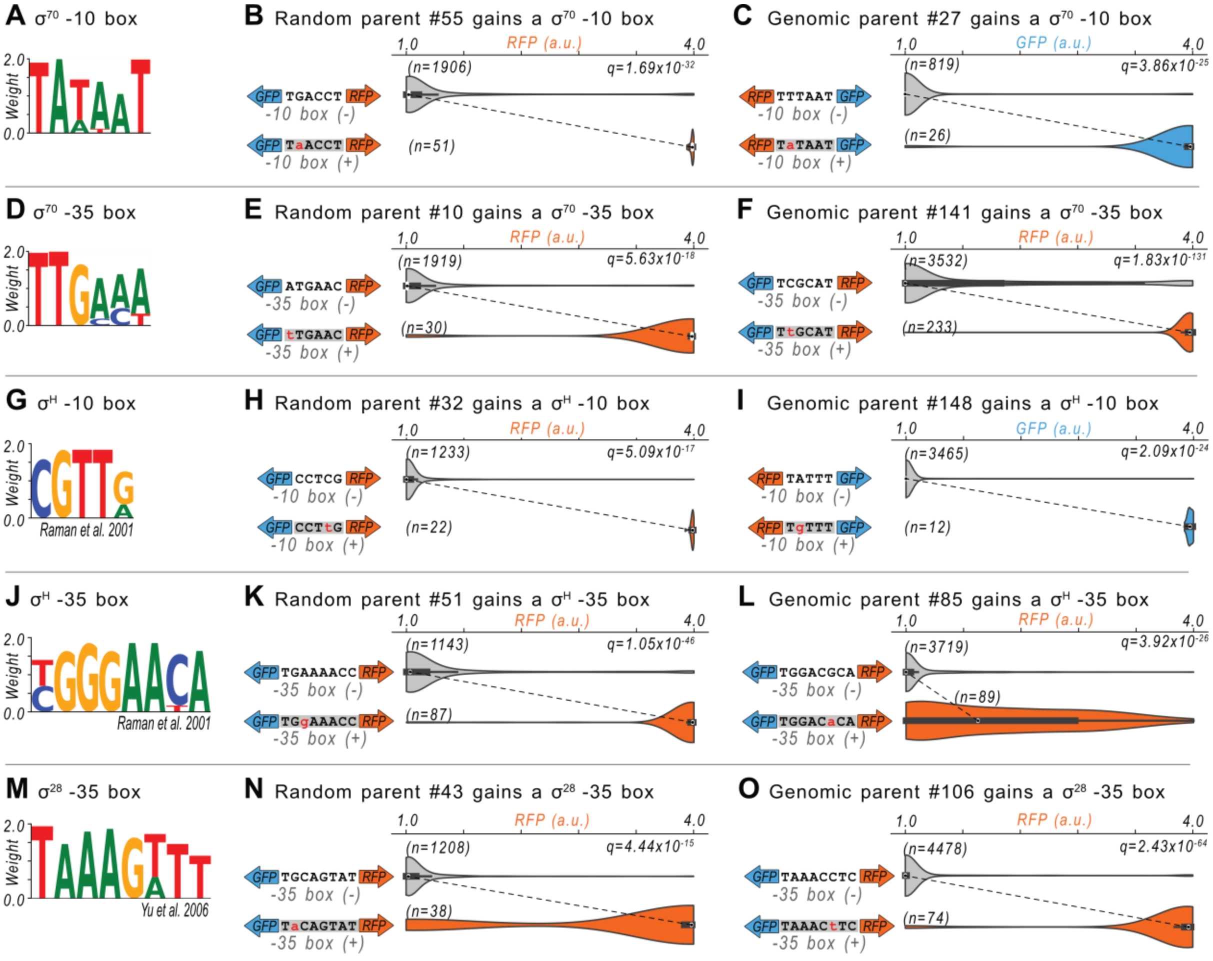

Fig. S9. Examples in which mutationally gained sites create promoter activity. (A) Sequence logo derived from a position-weight matrix (PWM, see Methods) of a -10 box for the sigma (σ) 70 housekeeping factor. The Logo was drawn by Logomaker(*61*). **(B)** Top left: a cropped representation of the DNA of a parent sequence. The arrows indicate the strand and orientation of the downstream reporter gene (teal, green fluorescent protein [GFP], orange, red fluorescent protein [RFP]). Top right: the distribution of fluorescence scores (a.u.) for daughter sequences encoding the sequence at the top left, which is not a predicted -10 box. Bottom left: analogous to the top left. The gray highlighted. DNA sequence corresponds to a gained PWM-predicted site for the transcription factor of interest. Bottom right: analogous to the top right, but for daughter sequences encoding the predicted site of interest at the bottom left. We test the null hypothesis that the two fluorescence distributions are the same with a two-tailed Mann-Whitney U test, and correct the calculated p-values for multiple hypothesis testing using the Benjamini-Hochberg procedure (*42*) with corresponding q-values. Panel (B) specifically corresponds to random parent #55 gaining a σ70 -10 box. **(C)** Analogous to (B) but for genomic #27 gaining a σ70 -10 box. **(D)** Analogous to (A) but for a σ70 -35 box. **(E)** Analogous to (B) but for random parent #10 gaining a σ70 -35 box. **(F)** Analogous to (B) but for genomic parent #141 gaining a σ70 -35 box. **(G)** Analogous to (A) but for a σH -10 box. **(H)** Analogous to (B) but for random parent #32 gaining a σH -10 box. **(I)** Analogous to (B) but for genomic parent #148 gaining a σH -10 box. **(J)** Analogous to (A) but for a σH -35 box. **(K)** Analogous to (B) but for random parent #51 gaining a σH -35 box. **(L)** Analogous to (B) but for genomic parent #85 gaining a σH -35 box. **(M)** Analogous to (A) but for a σ28 -35 box. **(N)** Analogous to (B) but for random parent #43 gaining a σ28 -35 box. **(O)** Analogous to (B) but for genomic parent #106 gaining a σ28 -35 box. See **Source Data**.

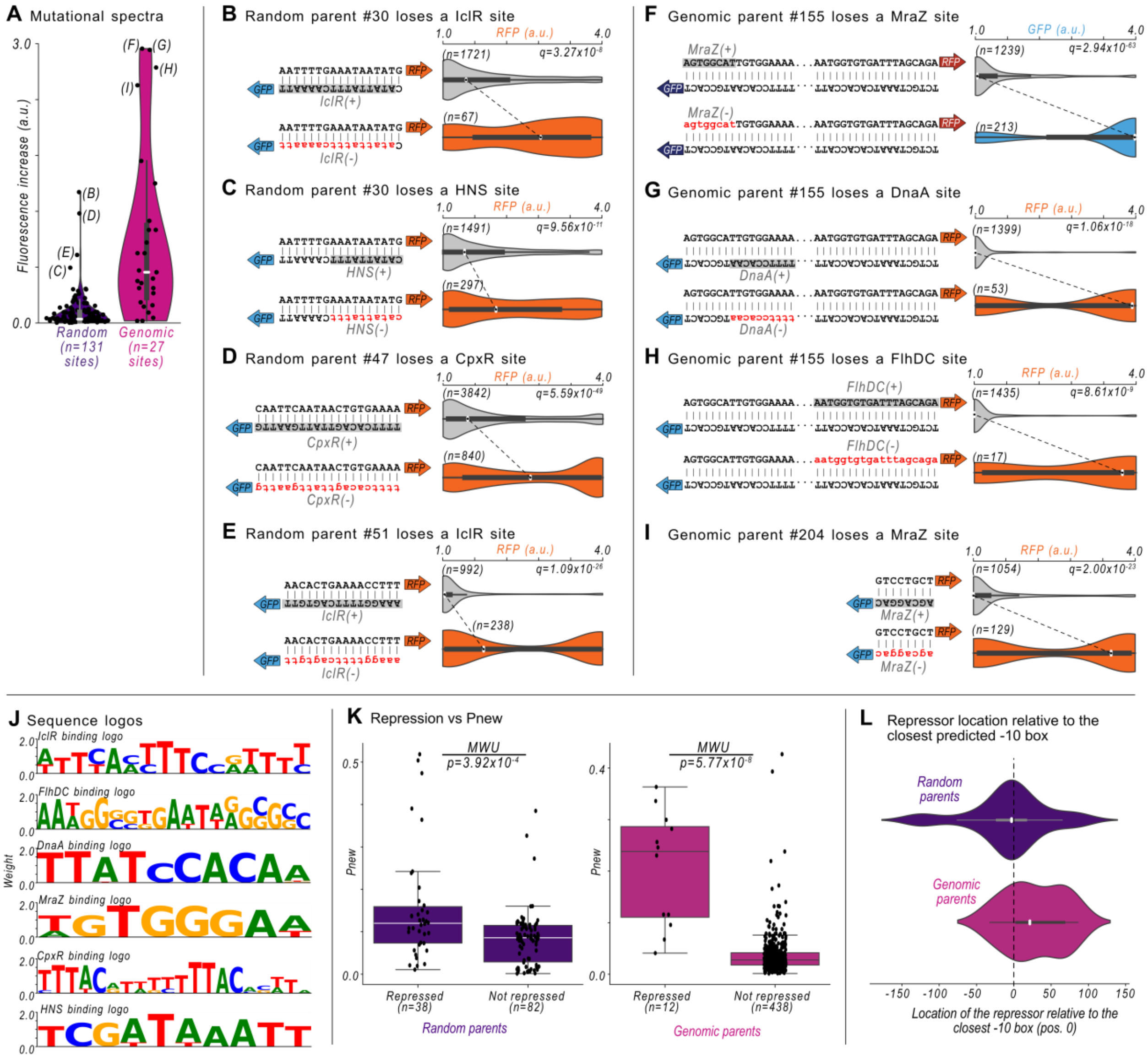

Fig. S10. Examples in which losing sites creates promoter activity. (A) Fluorescence increases (arbitrary units, a.u.) in parent sequences when a binding site is destroyed, for both random (left, purple) and genomic (right, magenta) parents. The white line shows the median, the gray box the interquartile range (IQR), whiskers span ±1 standard deviation, and the shaded area reflects a kernel density estimate. Black circles correspond to individual data points. Those annotated with letters are the subject of the remaining panels. **(B-I)** Top left: a cropped representation of the double-stranded DNA of a parent sequence. The arrows indicate the strand and orientation of the downstream reporter gene (teal, green fluorescent protein [GFP], orange, red fluorescent protein [RFP]). The DNA sequence highlighted in gray corresponds to a position-weight-matrix (PWM)-predicted binding site for the transcription factor of interest in the wild-type parent. Top right: the distribution of fluorescence scores (a.u.) for all daughter sequences encoding the predicted site of interest. Bottom left: analogous to the top left, but representing DNA sequences no longer encoding the predicted site of interest. Bottom right: analogous to the top right, but for daughter sequences no longer encoding the predicted site of interest. We test the null hypothesis that the fluorescence distributions are the same, using a two-tailed Mann-Whitney U test, and correcting the calculated p-values for multiple hypothesis testing using the Benjamini-Hochberg procedure (*42*) with corresponding q-values. See (A) for

details on violin plots. **(B)** Daughter sequences from random parent #30, and their RFP distributions with a IclR(+) and without the IclR(-) site of interest. **(C)** Analogous to B but for losing a HNS site. **(D)** Analogous to (B) but for random parent #47 and losing a CpxR site. **(E)** Analogous to (B) but for random parent #51 and losing a IclR site. **(F)** Analogous to (B) but for genomic parent #155 and losing a MraZ site. **(G)** Analogous to (B) but for genomic parent #155 and losing a DnaA site. **(H)** Analogous to (B) but for genomic parent #155 and losing a FlhDC site. **(I)** Analogous to (B) but for genomic parent #204 and losing a MraZ site. **(J)** Sequence logos derived from the position-weight matrices (PWMs, see Methods) IclR, DnaA, CpxR, FlhDC, MraZ, and HNS from RegulonDB (*38*). The Logos were drawn by Logomaker (*61*). **(K)** P_{new} - the probability that a parent sequence acquires promoter activity by mutation (fluorescence score ≥1.5 a.u., vertical axis) – vs whether the parent contains a repressing site (repressed) or not (not repressed). Left: random parents. Right: genomic parents. We test the null hypothesis that the P_{new} distributions are the same using a two-tailed Mann-Whitney U test (MWU, random parents: $p=3.92\times10^{-4}$, genomic parents: $p=5.77\times10^{-8}$). For each box plot, the central line corresponds to the median, the filled rectangle to the interquartile range (IQR), and the whiskers indicate ±1 standard deviation. **(L)** The distance each repressing site is to the closest PWM-predicted -10 box. Top: random parents. Bottom: genomic parents. Violin plots and box plots analogous to (A). See **Source Data**.

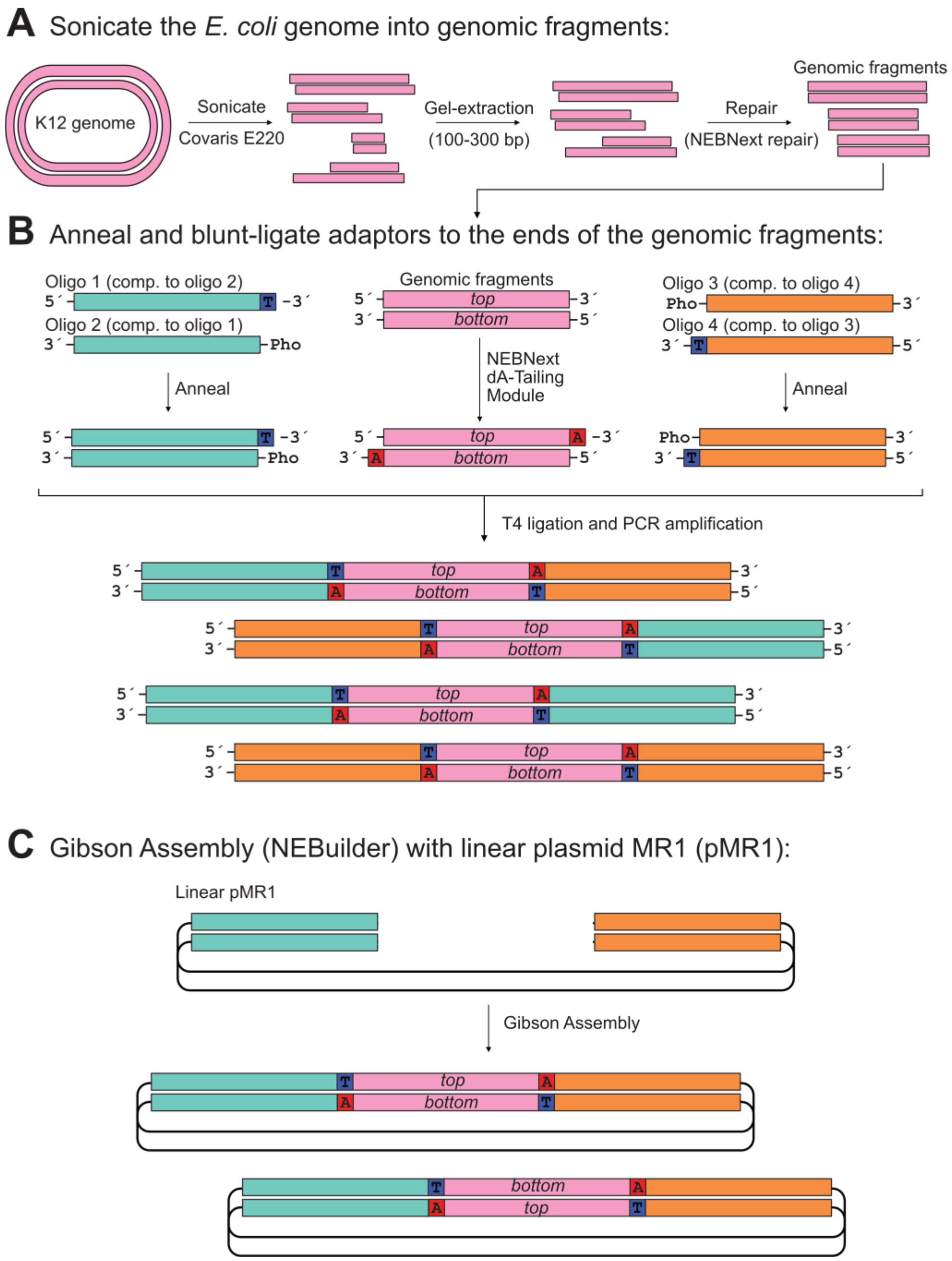

Fig. S11. Creating the wild-type genomic library. (A) We fragment the *E. coli* genome using an ultrasonicator, isolate sequences of 100-300 bps by gel extraction after electrophoresis, and repair the ends of the fragments using a repair kit. **(B)** Left: complementary ("comp") oligonucleotides ("oligos") are annealed. One of the oligos has a thymine (T) overhang on the 3'-end and the other a phosphate (Pho) on the 5'-end. The double-stranded product is homologous to the upstream region of plasmid MR1 (pMR1). Middle: We add an Adenine (A) to the 3'-end of the genomic fragments. Right: analogous to right, except the product is homologous to the downstream region of pMR1. Bottom: double-stranded products annealed using T4 ligation and polymerase chain reaction (PCR). **(C)** A Gibson Assembly reaction with the products from (B) and linearized copies of plasmid MR1 (pMR1) creates circularized plasmid libraries with genomic DNA in either orientation. See **Source Data**.

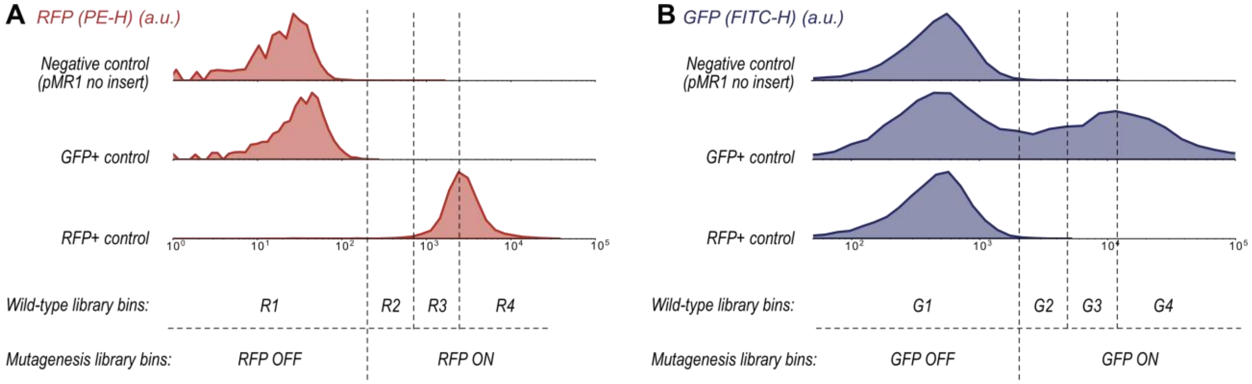

Fig. S12. Fluorescence-activated cell sorting boundaries. (A) Red fluorescent protein (RFP) readouts in the Phycoerythrin–Height (PE-H) channel for control plasmids. Top: negative control with the plasmid MR1 (pMR1) backbone and no insert. Middle: green fluorescent protein (GFP) positive control consists of the bba_J23110 promoter integrated into pMR1 oriented towards the GFP coding sequence. Bottom: RFP positive control consists of the bba_J23110 promoter integrated into pMR1 oriented towards the RFP coding sequence. The fluorescence distributions determine the fluorescence-activated cell sorting (FACS) bin boundaries, demarcated by vertical dashed lines. The wild-type libraries are sorted into bins R1, R2, R3, and R4. The mutagenesis libraries are sorted into bins RFP-OFF and RFP-ON. **(B)** Analogous to (A) but for Fluorescein Isothiocyanate-Height (FITC-H) and the same control plasmids. The wild-type libraries are sorted into bins G1, G2, G3, and G4. The mutagenesis libraries are sorted into bins GFP-OFF and GFP-ON. See **Methods** and **Source Data**.

Table S1.

A data frame (Microsoft Excel spreadsheet) containing putative promoter sequences from RegulonDB and their respective matches to the genomic sequences in our dataset. Available at: https://doi.org/10.5281/zenodo.18959816 and https://github.com/tfuqua95/random-genomic.

Data S1
Description: a data frame (csv format) containing the wild-type random DNA sequences and their respective fluorescence scores. Available at: https://doi.org/10.5281/zenodo.18959816 and https://github.com/tfuqua95/random-genomic.

Data S2
Description: a data frame (csv format) containing the wild-type genomic DNA sequences and their respective fluorescence scores. Available at: https://doi.org/10.5281/zenodo.18959816 and https://github.com/tfuqua95/random-genomic.

Data S3
Description: a data frame (csv format) containing the random parent mutagenesis library sequences and their respective fluorescence scores. Available at: https://doi.org/10.5281/zenodo.18959816 and https://github.com/tfuqua95/random-genomic.

Data S4
Description: a data frame (csv format) containing the genome parent mutagenesis library sequences and their respective fluorescence scores. Available at: https://doi.org/10.5281/zenodo.18959816 and https://github.com/tfuqua95/random-genomic.

Data S5
Description: a data frame (csv format) containing the associations between gaining or losing transcription factor or sigma factor binding sites and the change this has on fluorescence scores in the random parent sequences. Available at: https://doi.org/10.5281/zenodo.18959816 and https://github.com/tfuqua95/random-genomic.

Data S6
Description: a data frame (csv format) containing the associations between gaining or losing transcription factor or sigma factor binding sites and the change this has on fluorescence scores in the genome parent sequences. Available at: https://doi.org/10.5281/zenodo.18959816 and https://github.com/tfuqua95/random-genomic.

Data S7
Description: a data frame (Microsoft Excel spreadsheet) containing primer DNA sequences for polymerase chain reactions (PCRs) and molecular cloning. Available at: https://doi.org/10.5281/zenodo.18959816 and https://github.com/tfuqua95/random-genomic.

Source Data
Description: a data frame (Microsoft Excel spreadsheet) containing the values to recreate all figures in the manuscript. Available at: https://doi.org/10.5281/zenodo.18959816 and https://github.com/tfuqua95/random-genomic.
